# The RsfSR two-component system regulates SigF function by monitoring the state of the respiratory electron transport chain in *Mycobacterium smegmatis*

**DOI:** 10.1016/j.jbc.2024.105764

**Published:** 2024-02-16

**Authors:** Yuna Oh, Jeong-Il Oh

**Affiliations:** 1 Department of Integrated Biological Science, Pusan National University, Busan, Korea; 2 Microbiological Resource Research Institute, Pusan National University, Busan, Korea

**Keywords:** CHASE3, gene regulation, mycobacteria, *Mycobacterium smegmatis*, partner switching system, respiration, respiratory chain, SigF, sigma factor, two-component system

## Abstract

In *Mycobacterium smegmatis*, the transcriptional activity of the alternative sigma factor SigF is posttranslationally regulated by the partner switching system consisting of SigF, the anti-SigF RsbW1, and three anti-SigF antagonists (RsfA, RsfB, and RsbW3). We previously demonstrated that expression of the SigF regulon is strongly induced in the Δ*aa*_3_ mutant of *M. smegmatis* lacking the *aa*_3_ cytochrome *c* oxidase, the major terminal oxidase in the respiratory electron transport chain. Here, we identified and characterized the RsfSR two-component system involved in regulating the phosphorylation state of the major anti-SigF antagonist RsfB. RsfS (MSMEG_6130) is a histidine kinase with the cyclase/histidine kinase–associated sensing extracellular 3 domain at its N terminus, and RsfR (MSMEG_6131) is a receiver domain–containing protein phosphatase 2C–type phosphatase that can dephosphorylate phosphorylated RsfB. We demonstrated that phosphorylation of RsfR on Asp74 by RsfS reduces the phosphatase activity of RsfR toward phosphorylated RsfB and that the cellular abundance of the active unphosphorylated RsfB is increased in the Δ*aa*_3_ mutant relative to the WT strain. We also demonstrated that the RsfSR two-component system is required for induction of the SigF regulon under respiration-inhibitory conditions such as inactivation of the cytochrome *bcc*_1_ complex and *aa*_3_ cytochrome *c* oxidase, as well as hypoxia, electron donor-limiting, high ionic strength, and low pH conditions. Collectively, our results reveal a key regulatory element involved in regulating the SigF signaling system by monitoring the state of the respiratory electron transport chain.

The regulation of gene expression at the transcriptional level by alternative sigma factors is one of the important strategies for bacterial stress adaptation. Sigma factors reversibly bind to the core RNA polymerase (α_2_ββω) to allow it to bind to specific promoter sequences, thereby directing transcription of specific subsets of genes. *Mycobacterium smegmatis* and *Mycobacterium tuberculosis* among mycobacteria are known to have 28 and 13 sigma factor genes, respectively (1, 2, 3, 4, 5).

The SigF sigma factors, which are conserved in mycobacterial species, have been suggested to play a role in survival in the stationary growth phase, as well as in the adaptation to various stress conditions (1, 5, 6, 7, 8, 9, 10, 11, 12, 13, 14). Accordingly, expression of *sigF* in *M. tuberculosis* is induced under a variety of stress conditions, including acidic pH, oxidative stress, and nutrient depletion, as well as during the stationary phase and within cultured macrophages (7, 15, 16, 17, 18). In the case of *M. smegmatis*, deletion of *sigF* has been shown to lead to increased susceptibility to heat shock, oxidative stress, and acidic pH (9, 10), as well as the loss of carotenoid (isorenieratene) pigmentation (11, 12). Several lines of evidence have suggested that SigF is linked to the pathogenesis of *M. tuberculosis* (6, 7, 13, 14, 18, 19). In the mouse and guinea pig infection models, it has been demonstrated that *M. tuberculosis sigF* mutants were less virulent than their isogenic WT strain (13, 14). The genetic synteny of the *sigF* loci is well conserved in mycobacterial species. The anti-SigF genes [*usfX* (*rv3287c*) in *M. tuberculosis* and *rsbW1* (*MSMEG_1803*) in *M. smegmatis*] form operons with their downstream *sigF* genes (10, 20). The transcriptional activity of SigF is posttranslationally regulated by the partner switching system (PSS) consisting of SigF, antisigma factor, and antisigma factor antagonists (anti-anti-sigma factors) (Fig. 1) (11, 21, 22, 23). The anti-SigF binds to SigF and prevents SigF from its association with the core RNA polymerase, abolishing the transcriptional activity of SigF (11, 23). The functionality of anti-SigF is under the negative control of the anti-SigF antagonists RsfA (Rv1365c in *M. tuberculosis* and MSMEG_1786 in *M. smegmatis*) and RsfB (Rv3687c in *M. tuberculosis* and MSMEG_6127 in *M. smegmatis*) (11, 21, 22, 24, 25). The functionality of RsfA is controlled by the redox state of its two cysteine residues (Cys73 and Cys109 in *M. tuberculosis*) (25). RsfB contains the sulfate transporter and antisigma antagonist (STAS) domain that can be phosphorylated at a specific serine residue (Ser61 in *M. tuberculosis* and Ser63 in *M. smegmatis*), and it is inactivated upon phosphorylation of the serine residue (21, 22). RsfB was demonstrated to be the major anti-SigF antagonist in *M. smegmatis* (22). *M. smegmatis* has three RsbW homologs [RsbW1, RsbW2 (MSMEG_6129), and RsbW3 (MSMEG_1787)] (22). Among them, only RsbW1 was demonstrated to be able to bind to SigF, serving as an anti-SigF (22). RsbW2 was demonstrated to be unable to interact with SigF directly but it serves as a Ser/Thr protein kinase (STPK) that phosphorylates the Ser63 residue of RsfB, leading to inactivation of RsfB (22). RsbW3 with high homology to RsbW1 functions as an anti-SigF antagonist rather than anti-SigF by forming the RsbW1-RsbW3 heterodimer (22), which suggests that three anti-SigF antagonists (RsfA, RsfB, and RsbW3) and one anti-SigF (RsbW1) constitute the SigF PSS in *M. smegmatis*. In the case of *M. tuberculosis*, the proteins corresponding to RsbW2 and RsbW3 have not been identified. The Rv1364c protein, which was suggested to be a component of the SigF PSS in *M. tuberculosis*, is a multidomain protein consisting of the Per-Arnt-Sim, protein phosphatase 2C (PP2C) phosphatase, gyrase, Hsp90, histidine kinase, MutL kinase, and STAS domains (24, 26, 27). Rv1364c was shown to interact with SigF *in vitro*, suggesting that it might act as an anti-SigF along with UsfX (26). Although Rv1364c was demonstrated to have both STPK and phosphatase activities to autophosphorylate and dephosphorylate the serine residue (Ser600) in its STAS domain (26, 27), its involvement in phosphorylation and dephosphorylation of RsfB has yet to be elucidated.

**Figure 1:**
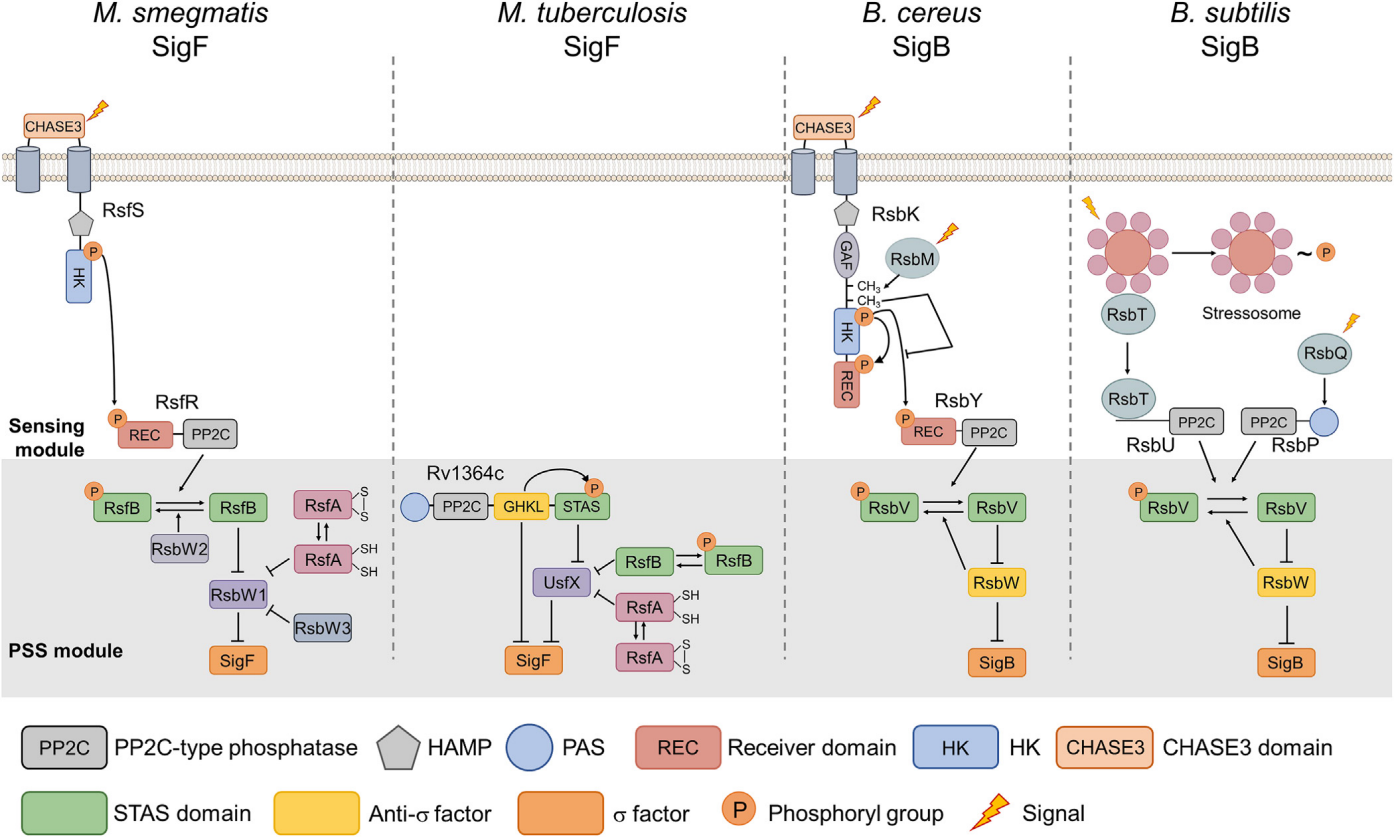
**Schematic illustration of the SigF and SigB PSSs and their associated sensing modules in *Mycobacterium smegmatis, Mycobacterium tuberculosis, Bacillus cereus,* and *Bacillus subtilis*.** The phosphoryl group is denoted by the encircled "P." RsbW of the *Bacillus* species acts as both an antisigma factor and protein kinase phosphorylating the anti-SigB antagonist RsbV. CHASE3, cyclases/histidine kinases–associated sensory extracellular 3 domain; HAMP, histidine kinases, adenyl cyclases, methyl-accepting proteins and phosphatases; HK, histidine kinase domain; PAS, Per-Arnt-Sim domain; PP2C, protein phosphatase 2C domain; PSS, partner switching system; REC, receiver domain; STAS, sulfate transporter and antisigma antagonist domain.

Several independent studies have shown that the SigF regulon is induced in mycobacteria under respiration-inhibitory conditions. It has been reported that expression of the SigF regulon is upregulated under hypoxic conditions in *M. tuberculosis* and *M. smegmatis* (15, 28, 29, 30). Our comparative RNA-seq analysis of the WT strain of *M. smegmatis* and its isogenic Δ*aa*_3_ mutant strain lacking the *aa*_3_ cytochrome *c* oxidase, the major terminal oxidase of the respiratory electron transport chain (ETC), revealed that expression of the SigF regulon is strongly induced in the Δ*aa*_3_ mutant in an RsfB-dependent way (22). However, it remains elusive how the phosphorylation state of RsfB in mycobacteria is regulated by reflecting the functional state of the respiratory ETC.

In this study, we identified and characterized a new two-component system (TCS) that is involved in the regulation of the phosphorylation state of RsfB. The identified TCS is composed of the receiver domain–containing PP2C-type phosphatase RsfR (MSMEG_6131) and the RsfS (MSMEG_6130) histidine kinase (HK). RsfR serves as a phosphatase that dephosphorylates RsfB. RsfS is a putative membrane–associated HK with the cyclase/histidine kinase–associated sensing extracellular 3 (CHASE3) domain in its N-terminal sensory domain. We here suggest the possibility that the membrane-associated RsfS HK is a sensor kinase that recognizes the state of the ETC and is responsible for the induction of the SigF regulon through the regulation of RsfR phosphatase activity under respiration-inhibitory conditions.

## Results

### RsfR and RsfS are required for an increase in SigF functionality in the Δ*aa*_3_ mutant of *M. smegmatis*

**Figure 2:**
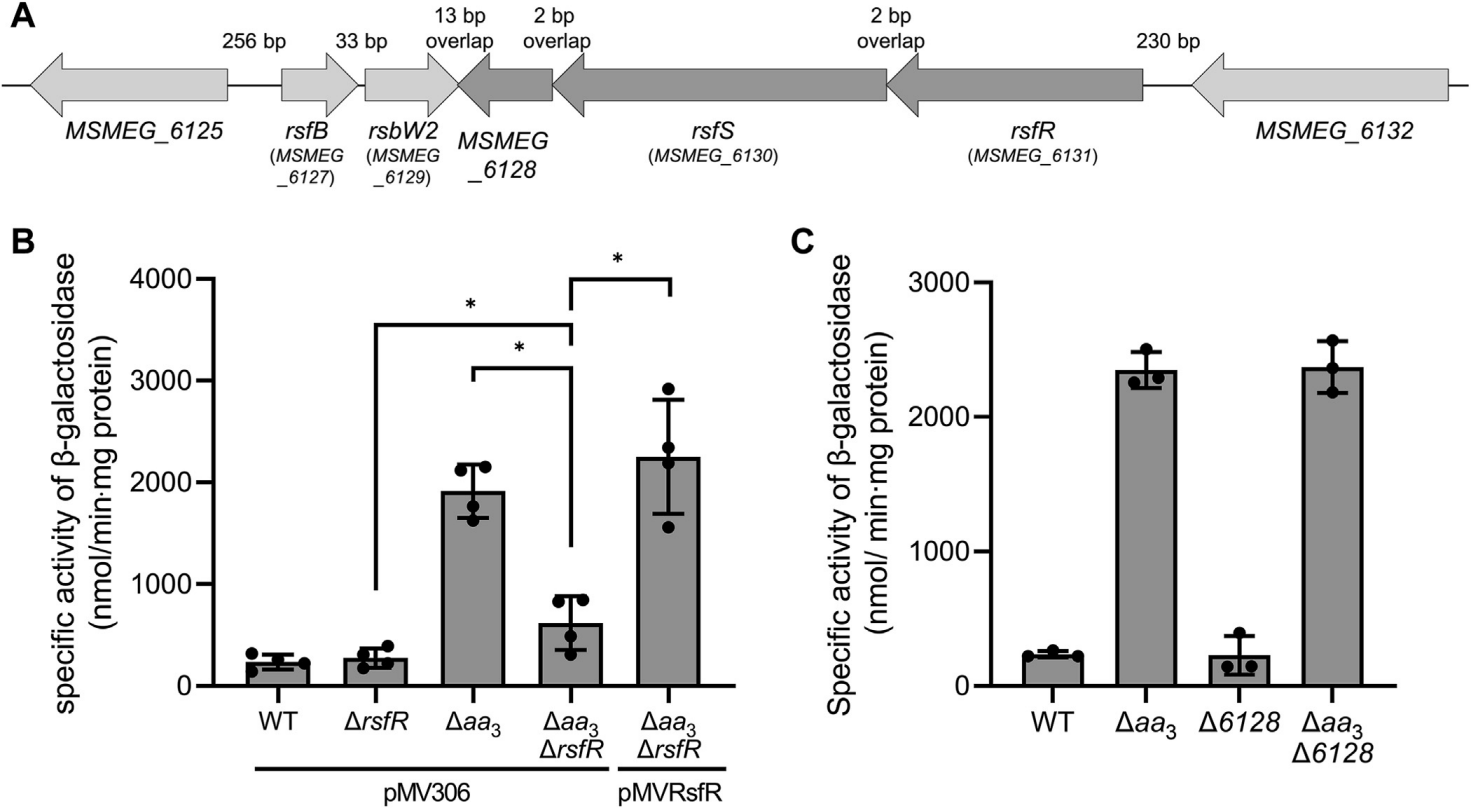
**Effects of inactivation of *rsfR* or *MSMEG_6128* on expression of *MSMEG_1777* in *Mycobacterium smegmatis*.***A*, genetic organization of the *rsfB* locus of *M. smegmatis* mc^2^ 155. The *arrows* denote the ORFs and their transcriptional direction. The locus tag numbers of the genes are presented in *parentheses* below the gene names. The lengths of the intergenic and overlapping regions are given as the nucleotide numbers above the schematic diagram. *B*, expression of *MSMEG_1777* in the WT, Δ*aa*_3_, Δ*rsfR*, and Δ*aa*_3_Δ*rsfR* strains of *M. smegmatis*. The *M. smegmatis* strains harboring both the empty vector pMV306 and the *MSMEG_1777*::*lacZ* translational fusion plasmid pNCII1777 were used in the experiments. For complementation of the Δ*aa*_3_Δ*rsfR* mutant, pMVRsfR (a pMV306-derived plasmid carrying the intact *rsfR* gene and its own promoter) was used in place of pMV306. *C*, expression of *MSMEG_1777* in the WT, Δ*aa*_3_, Δ*6128*, and Δ*aa*_3_Δ*6128* mutant strains of *M. smegmatis*. The *M. smegmatis* strains harboring pNCII1777 were grown aerobically to an *A*_600_ of 0.45 to 0.5 in 7H9-glucose medium. Expression of *MSMEG_1777* was quantified by determining β-galactosidase activity in the strains. All values are the means of the results from four and three biological replicates for *panel B* and *panel C*, respectively. The error bars indicate the SDs. ∗*p* < 0.05.

We previously demonstrated that inactivation of the *aa*_3_ cytochrome *c* oxidase in *M. smegmatis* by mutation leads to both a decrease in the respiration rate by approximately 50% under aerobic culture conditions and a significant increase in expression of the SigF regulon (22, 31). We also suggested that RsfB, the major anti-SigF antagonist in *M. smegmatis*, mediates induction of the SigF regulon in the Δ*aa*_3_ mutant lacking the *aa*_3_ cytochrome *c* oxidase (22). The *rsfB* gene forms an operon with its downstream *rsbW2* gene encoding the STPK that phosphorylates RsfB to inactivate it (22). To search for a phosphatase that dephosphorylates phosphorylated RsfB, we first examined whether there is a gene encoding a protein containing a phosphatase domain in the upstream and downstream regions of the *rsfB*-*rsbW2* operon. Downstream of *rsbW2* is a putative operon consisting of three ORFs (*MSMEG_6131*, *MSMEG_6130*, and *MSMEG_6128*) with the transcriptional orientation opposite to *rsbW2* (Fig. 2*A*). *MSMEG_6130* encodes a HK with the CHASE3 domain flanked by two putative transmembrane α-helices at its N-terminal domain, and its neighboring genes *MSMEG_6128* and *MSMEG_6131* encode proteins with a receiver domain at their N-terminal domains. Sequence analysis revealed that MSMEG_6131 contains a PP2C phosphatase domain at its C-terminal domain, while MSMEG_6128 has a helix-turn-helix motif for a DNA-binding domain. This finding indicates the possibility that MSMEG_6130 constitutes a TCS with either MSMEG_6128 or MSMEG_6131 or both. From now on, we refer to MSMEG_6130 and MSMEG_6131 as RsfS and RsfR, respectively.

To examine whether RsfR is involved in the regulation of the SigF PSS, we measured the expression level of *MSMEG_1777* in the WT and mutant strains of *M. smegmatis* (WT, Δ*aa*_3_, Δ*rsfR*, Δ*aa*_3_Δ*rsfR*) using the *MSMEG_1777::lacZ* translational fusion plasmid pNCII1777. Since it had been reported that *MSMEG_1777* is strictly dependent on SigF for its expression (22), it was used as a reporter gene to measure the transcriptional activity of SigF. As shown in Figure 2*B*, the expression level of *MSMEG_1777* in the Δ*aa*_3_ mutant grown aerobically was increased by 8-fold relative to that in the WT strain grown under the same conditions, which is consistent with our previous report (22). The expression level of *MSMEG_1777* was significantly reduced by deletion of *rsfR* in the background of the Δ*aa*_3_ mutant (Δ*aa*_3_Δ*rsfR*) compared to that in the parental Δ*aa*_3_ strain, while the WT and Δ*rsfR* strains exhibited basal expression of *MSMEG_1777*. Compared to the Δ*rsfR* mutant, the Δ*aa*_3_Δ*rsfR* mutant still showed a slightly higher level of *MSMEG_1777* expression. This result indicates that RsfR plays a major role in induction of *MSMEG_1777* expression in the Δ*aa*_3_ mutant and that there is another mechanism by which expression of *MSMEG_1777* is slightly increased in an RsfR-independent way by inactivation of the *aa*_3_ oxidase. The ectopic expression of the intact *rsfR* gene in the Δ*aa*_3_Δ*rsfR* mutant using pMVRsfR restored the expression level of *MSMEG_1777* to that in the Δ*aa*_3_ strain with the empty vector pMV306, conﬁrming that the severely impaired expression of *MSMEG_1777* in the Δ*aa*_3_Δ*rsfR* mutant is the result of *rsfR* inactivation.

To examine whether MSMEG_6128 is also implicated in the regulation of the SigF PSS, we determined the expression level of *MSMEG_1777* in the WT, Δ*6128*, Δ*aa*_3_, and Δ*aa*_3_Δ*6128* strains carrying pNCII1777 (Fig. 2*C*). The deletion of *MSMEG_6128* in the WT and Δ*aa*_3_ mutant strains (Δ*6128* and Δ*aa*_3_Δ*6128*) did not change the expression level of *MSMEG_1777* compared to the parental strains (WT and Δ*aa*_3_), indicating that MSMEG_6128 is not involved in the regulation of the SigF PSS.

**Figure 3:**
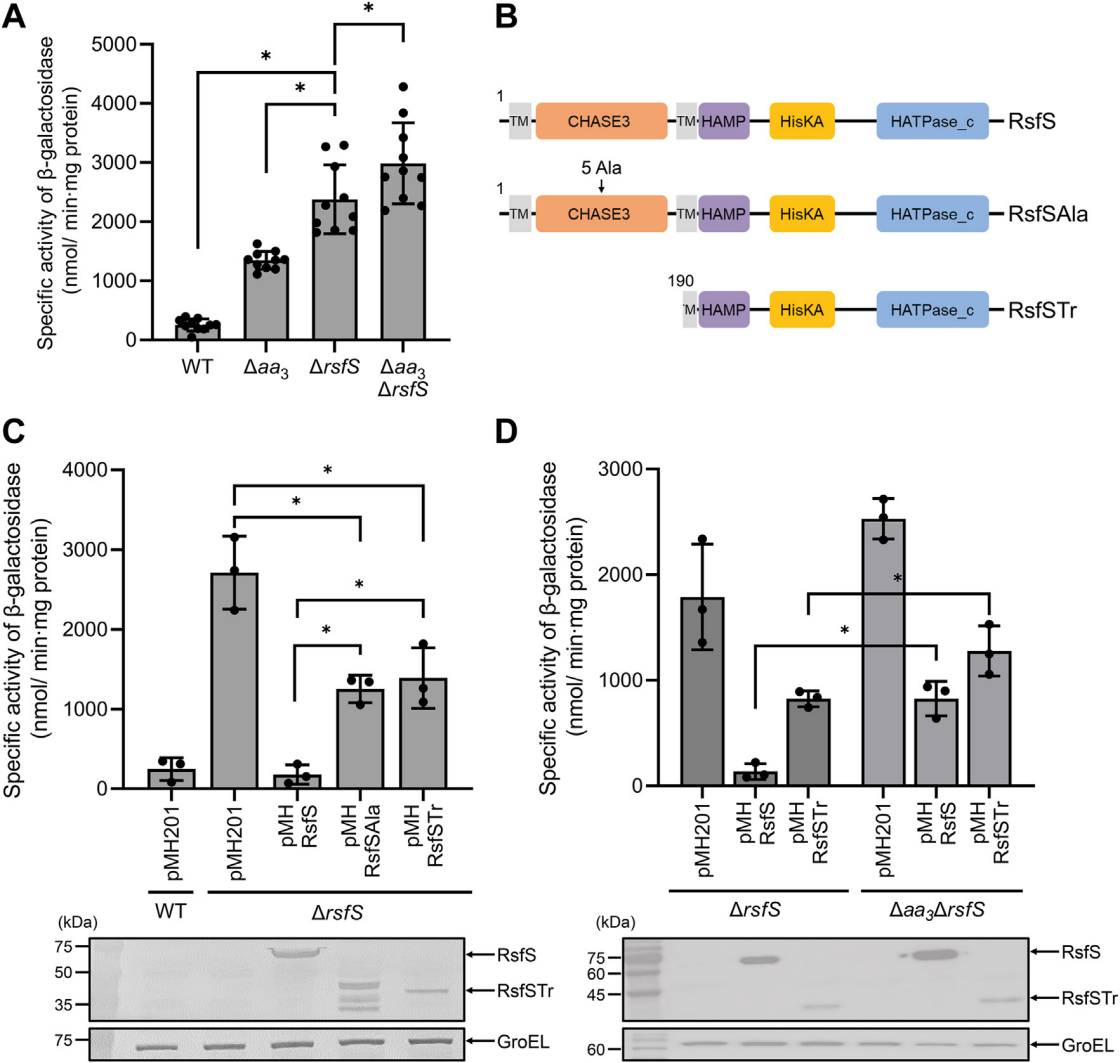
**Effects of inactivation of *rsfS* on expression of *MSMEG_1777* in *Mycobacterium smegmatis*.***A*, expression of *MSMEG_1777* in the WT, Δ*aa*_3_, Δ*rsfS*, and Δ*aa*_3_Δ*rsfS* strains of *M. smegmatis*. The *M. smegmatis* strains harboring pNCII1777 were grown aerobically to an *A*_600_ of 0.45 to 0.5 in 7H9-glucose medium. *B*, *schematic diagram* depicting the domain structure of the WT and mutant forms of RsfS. RsfSAla contains five consecutive Ala residues in the middle of the CHASE3 domain (between amino acids 108 and 109), and RsfSTr is an N terminally truncated RsfS (amino acids 190–525). *C*, complementation analysis of the Δ*rsfS* mutant strain with the WT (RsfS) and mutant (RsfSAla and RsfSTr) forms of the *rsfS* gene. The complementation test was performed by the introduction of pMHRsfS, pMHRsfSAla, or pMHRsfSTr into the Δ*rsfS* mutant with pNCII1777. The WT and Δ*rsfS* strains with both pNCII1777 and the empty vector pMH201 were included as controls. The *M. smegmatis* strains were grown aerobically to an *A*_600_ of 0.45 to 0.5 in 7H9-glucose medium in the presence of 0.1% acetamide. Crude extracts (10 μg for detection of RsfS and its variants; 5 μg for GroEL detection) were subjected to Western blotting analysis to detect the C terminally 2B8-tagged RsfS and GroEL. The protein level of GroEL was determined as a loading control. *D*, complementation analysis of the Δ*rsfS* and Δ*aa*_3_Δ*rsfS* mutant strains with the WT and truncated forms of the *rsfS* gene. The complementation test was performed by the introduction of pMHRsfS or pMHRsfSTr into the mutants with pNCII1777. The Δ*rsfS* and Δ*aa*_3_Δ*rsfS* strains with both pNCII1777 and pMH201 were included as controls. The *M. smegmatis* strains were grown aerobically to an *A*_600_ of 0.45 to 0.5 in 7H9-glucose medium in the presence of 0.1% acetamide. Western blotting analysis was performed in the same way as *panel C*. Cell crude extracts were used to determine β-galactosidase activity. All values are the means of the results from ten biological replicates for *panel A* and three biological replicates for *panel C* and *panel D*. The error bars indicate the SDs. ∗*p* < 0.05. CHASE3, cyclase/histidine kinase–associated sensing extracellular 3.

Since *rsfS* belongs to the putative *rsfR*-*rsfS*-*MSMEG_6128* operon, it is likely that RsfS is involved in the regulation of the SigF PSS. To examine this possibility, we determined the expression level of *MSMEG_1777* in the WT, Δ*rsfS*, Δ*aa*_3_, and Δ*aa*_3_Δ*rsfS* strains carrying pNCII1777 (Fig. 3*A*). Expression of *MSMEG_1777* was derepressed in the Δ*rsfS* strain by 9.3-fold relative to that in the WT strain when the strains were grown aerobically. Inactivation of the *aa*_3_ oxidase in the background of the Δ*rsfS* mutant (Δ*aa*_3_Δ*rsfS*) led to only a slight increase in *MSMEG_1777* expression compared to the parental Δ*rsfS* mutant. This result suggests that RsfS is involved in repression of the SigF regulon under normal respiration conditions. We next performed complementation analysis of the Δ*rsfS* mutant with pMHRsfS carrying the intact *rsfS* gene. At the same time, we examined whether the truncated RsfS (RsfSTr), consisting only of the kinase domain (amino acids 190–525), and the mutant form of RsfS (RsfSAla) in which a string of five alanine residues is inserted in the middle of the CHASE3 domain (between amino acids 108 and 109) to disrupt the CHASE3 domain are functional (Fig. 3*B*). For this experiment, the pMH201-derived pMHRsfS, pMHRsfSAla, and pMHRsfSTr plasmids were constructed to express RsfS, RsfSAla, and RsfSTr from an acetamide-inducible promoter, respectively, and the expression level of *MSMEG_1777* was determined in the Δ*rsfS* strains with the pMH201 derivatives. We also included the WT and Δ*rsfS* mutant strains with the empty pMH201 vector in the experiment as positive and negative controls, respectively. As shown in Figure 3*C*, the ectopic expression of *rsfS* from pMHRsfS led to complementation of the Δ*rsfS* mutant, while the introduction of the genes encoding pMHRsfSAla and pMHRsfSTr into the Δ*rsfS* mutant resulted in partial complementation. Western blotting analysis showed that RsfS and RsfSTr with the expected molecular weight were expressed in the Δ*rsfS* strains with pMHRsfS and pMHRsfSTr, respectively. Intriguingly, expressed RsfSAla was detected as several bands smaller than expected, indicating the instability of RsfSAla. To examine whether RsfSTr is functional to induce expression of *MSMEG_1777* in the Δ*aa*_3_ mutant, we comparatively determined the expression level of *MSMEG_1777* in the aerobically grown Δ*rsfS* and Δ*aa*_3_Δ*rsfS* mutants that harbor pMHRsfSTr. We included the Δ*rsfS* and Δ*aa*_3_Δ*rsfS* mutant strains with pMH201 or pMHRsfS in the experiment as controls (Fig. 3*D*). Expression of *MSMEG_1777* was 6.1-fold increased in the Δ*aa*_3_Δ*rsfS* strain with pMHRsfS relative to the Δ*rsfS* strain with pMHRsfS. In contrast, the Δ*aa*_3_Δ*rsfS* strain with pMHRsfSTr exhibited a marginal (1.5-fold) increase in *MSMEG_1777* expression compared to the Δ*rsfS* strain with pMHRsfSTr. This induction fold (1.5-fold) is similar to the induction fold of *MSMEG_1777* expression observed for the Δ*aa*_3_Δ*rsfS* strain with pMH201 relative to the Δ*rsfS* strain with pMH201, which suggests that RsfSTr lost its ability to induce expression of *MSMEG_1777* in response to inhibition of the respiratory ETC.

### RsfS and RsfR form a TCS

**Figure 4:**
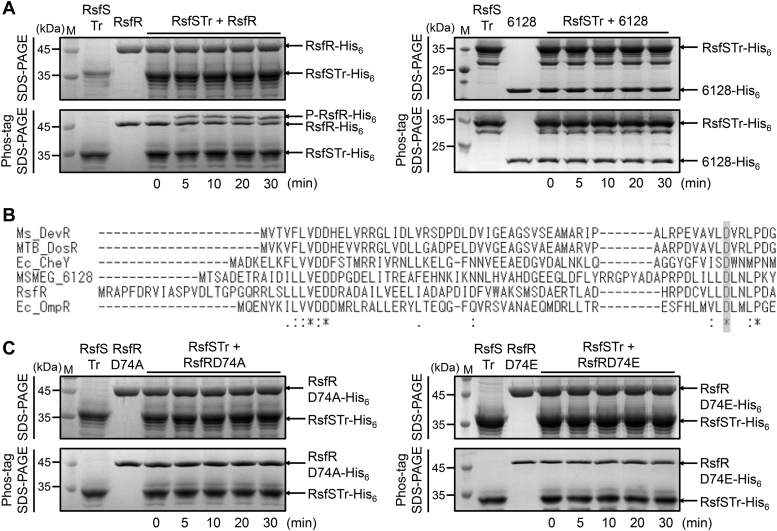
**RsfS-dependent phosphorylation of RsfR and identification of the phosphorylation residue in RsfR.***A*, phosphotransfer from phosphorylated RsfSTr to RsfR and MSMEG_6128. 550 pmol of partially purified RsfSTr was autophosphorylated in the reaction mixture [300 mM Tris-HCl (pH 8), 50 mM KCl, 10 mM MgCl_2_, and 1 mM ATP] for 30 min at 30 °C. Following the addition of either 154 pmol of purified RsfR or 330 pmol of purified MSMEG_6128 to the autophosphorylation reaction mixture (the total reaction volume is 22 μl), the phosphotransfer reactions were performed at 30 °C. The reactions were stopped at the indicated time points by the addition of 11 μl of 3x gel-loading buffer. Fifteen microliters each of the stopped reactions was subjected to SDS-PAGE (*upper gel*) and 75 μM Mn^2+^-Phos-tag SDS-PAGE (*lower gel*). The gels were stained with CBB. The bands of His_6_-tagged RsfSTr (RsfSTr-His_6_), MSMEG_6128 (6128-His_6_), unphosphorylated RsfR (RsfR-His_6_), and phosphorylated RsfR (P-RsfR-His_6_) are indicated by the *arrows*. *B*, identification of the phosphorylation site in RsfR. Multiple alignment of the receiver domains of RsfR and MSMEG_6128 with those of several response regulators from *Escherichia coli* (Ec)*, Mycobacterium tuberculosis* (MTB), and *Mycobacterium smegmatis* (Ms) was generated using ClustalW. The *asterisks and colons* denote the conserved and conservatively substituted amino acid residues, respectively. The known phosphorylation residues of the response regulators and the corresponding residues of RsfR and MSMEG_6128 are highlighted in the *gray background*. *C*, effect of D74A and D74E mutations on phosphorylation of RsfR by RsfSTr. Phosphotransfer reactions using purified RsfSTr and mutant forms of RsfR (D74A and D74E) were performed in the same way as *panel A*. CBB, Coomassie brilliant blue; M, molecular weight marker lanes; RsfSTr, truncated RsfS.

From the adjacent genetic location of *rsfS* and *rsfR* and the finding that both gene products are involved in the regulation of the SigF PSS, we assumed that RsfS and RsfR constitute a TCS. To examine this assumption, we carried out *in vitro* phosphorylation assay using purified RsfSTr and RsfR to determine whether RsfS can phosphorylate RsfR. As shown in Figure 4*A*, phosphotransfer from autophosphorylated RsfSTr to RsfR occurred as judged by the appearance of the retarded RsfR bands on the Phos-tag SDS-PAGE gel. To identify the amino acid residue of RsfR that is phosphorylated by RsfS, we performed multiple sequence alignment of the receiver domains of RsfR and other response regulators (Fig. 4*B*), which allowed us to assume that Asp74 in RsfR is the residue phosphorylated by RsfS. To confirm this assumption, *in vitro* phosphorylation assay was performed using the mutant forms (D74A and D74E) of RsfR and RsfSTr. As shown in Figure 4*C*, RsfSTr phosphorylated neither RsfRD74A nor RsfRD74E, indicating that it is Asp74 in RsfR that is phosphorylated by RsfS. We also examined whether MSMEG_6128 is phosphorylated by RsfSTr. Unlike RsfR, MSMEG_6128 was not phosphorylated by RsfSTr, indicating that RsfS forms a TCS with RsfR, not with MSMEG_6128.

### The RsfSR TCS is responsible for controlling the phosphorylation state of RsfB

**Figure 5:**
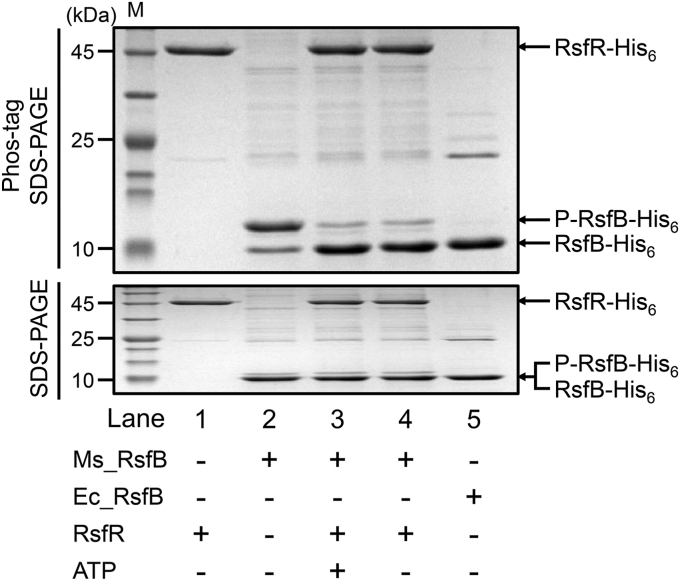
**Phosphatase activity of RsfR acting on phosphorylated RsfB.***In vitro* dephosphorylation assay using purified RsfR and RsfB (*lanes 3* and *4*). 800 pmol of phosphorylated RsfB purified from *Mycobacterium smegmatis* (Ms_RsfB) was mixed with 100 pmol of purified RsfR in the reaction mixture [300 mM Tris-HCl (pH 8.0), 50 mM KCl, and 10 mM MgCl_2_] (the total reaction volume is 22 μl). The dephosphorylation reactions were performed for 30 min at 30 °C in the presence and absence of 1 mM ATP and stopped by the addition of 11 μl of 3x loading buffer. Subsequently, 15 μl each of the stopped reactions was subjected to 50 μM Mn^2+^-Phos-tag SDS-PAGE (*upper gel*) and SDS-PAGE (*lower gel*). The gels were stained with CBB. The bands of His_6_-tagged RsfR (RsfR-His_6_), unphosphorylated RsfB (RsfB-His_6_), and phosphorylated RsfB (P-RsfB-His_6_) are indicated by the *arrows*. Purified RsfR and RsfB purified from *M. smegmatis* (Ms_RsfB) and *Escherichia coli* (Ec_RsfB) were loaded into *lane 1, lane 2, and lane 5* to indicate the bands of RsfR, phosphorylated RsfB, and unphosphorylated RsfB, respectively. CBB, Coomassie brilliant blue; M, molecular weight marker lanes.

Since RsfR contains a PP2C phosphatase domain, we examined whether RsfR serves as a phosphatase that dephosphorylates phosphorylated RsfB in *M. smegmatis*. To ascertain this possibility, we performed *in vitro* dephosphorylation assay using purified RsfR and RsfB. As shown in Figure 5, the major fraction of RsfB purified from *M. smegmatis* (Ms_RsfB) was found to be present in a phosphorylated form as judged by Phos-tag SDS-PAGE (lane 2), whereas RsfB purified from *Escherichia coli* was found to be exclusively in the unphosphorylated state (lane 5). The mixing and incubation of Ms_RsfB with RsfR led to conversion of most phosphorylated Ms_RsfB to the unphosphorylated form regardless of the presence or absence of ATP (lanes 3 and 4), indicating that RsfR has the phosphatase activity to dephosphorylate phosphorylated RsfB in an ATP-independent way.

**Figure 6:**
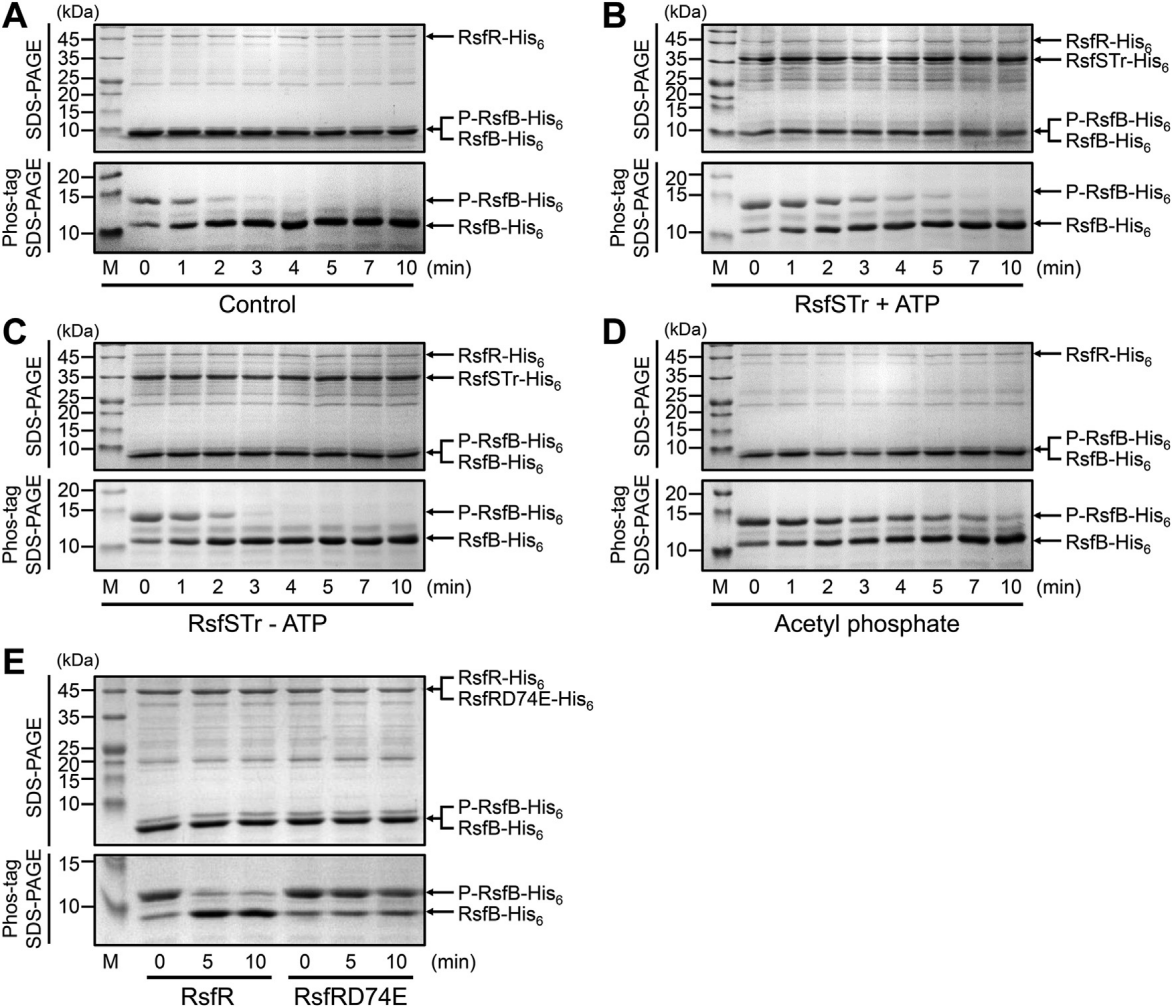
**Effect of phosphorylation of RsfR on its phosphatase activity.***A*, dephosphorylation of RsfB by unphosphorylated RsfR (control). 22 pmol of purified RsfR was mixed with 440 pmol of RsfB purified from *Mycobacterium smegmatis* in the reaction mixture [300 mM Tris-HCl (pH 8.0), 50 mM KCl, 10 mM MgCl_2_, and 1 mM ATP] (the total reaction volume is 22 μl). The dephosphorylation reactions were performed at 30 °C and terminated at the indicated time points by the addition of 11 μl of 3x gel-loading buffer. To examine the phosphorylation extent of RsfB, 15 μl each of the stopped reactions was subjected to SDS-PAGE (*upper gel*) and 50 μM Mn^2+^-Phos-tag SDS-PAGE (*lower gel*). *B* and *C*, dephosphorylation of RsfB by RsfR pretreated with RsfSTr. After 22 pmol of RsfR was incubated with 200 pmol of partially purified RsfSTr in the reaction mixture with 1 mM ATP (*B*) or without ATP (*C*) for 30 min at 30 °C, 440 pmol of RsfB was added to the reaction mixtures. The dephosphorylation reactions and Phos-tag SDS-PAGE were performed in the same way as described in *panel A*. *D*, dephosphorylation of RsfB by RsfR phosphorylated by acetyl phosphate. Following 22 pmol of RsfR was incubated in the ATP-free reaction mixture containing 40 mM acetyl phosphate for 30 min at 30 °C, 440 pmol of RsfB was added to the reaction mixture. The dephosphorylation reactions and Phos-tag SDS-PAGE were performed in the same way as described in *panel A*. *E*, dephosphorylation of RsfB by unphosphorylated RsfR or RsfRD74E. 66 pmol of purified RsfR or RsfRD74E was mixed with 440 pmol of RsfB purified from *M. smegmatis* in the reaction mixture. The dephosphorylation reactions were allowed to proceed at 30 °C and terminated at the indicated time points. The phosphorylation extent of RsfB was determined using Phos-tag SDS-PAGE. The gels were stained with CBB. The bands of RsfSTr (RsfSTr-His_6_), RsfR (RsfR-His_6_), RsfRD74E (RsfRD74E-His_6_), unphosphorylated RsfB (RsfB-His_6_), and phosphorylated RsfB (P-RsfB-His_6_) are indicated by the *arrows*. CBB, Coomassie brilliant blue; M, molecular weight marker lanes.

We next performed *in vitro* dephosphorylation assay to determine the effect of phosphorylation of RsfR on its phosphatase activity toward phosphorylated RsfB (Fig. 6). Since RsfSTr was shown to phosphorylate RsfR on Asp74 (Fig. 4), we used RsfSTr to phosphorylate RsfR. When Ms_RsfB was mixed with unphosphorylated RsfR, phosphorylated Ms_RsfB was decreased over time with a concurrent increase in unphosphorylated Ms_RsfB on the Phos-tag SDS-PAGE gel, which indicates dephosphorylation of Ms_RsfB by RsfR (Fig. 6*A*). When RsfR was preincubated with RsfSTr in the presence of ATP, the dephosphorylation rate of Ms_RsfB by RsfR was shown to be noticeably slower than when RsfR was not preincubated with RsfSTr (Fig. 6*B*). In contrast, preincubation of RsfR with RsfSTr in the absence of ATP did not result in slower dephosphorylation of Ms_RsfB (Fig. 6*C*). These results indicate that phosphorylation of RsfR results in a decrease in its phosphatase activity. Acetyl phosphate is a well-known direct phosphodonor for response regulators. As shown in Figure 6*D*, phosphorylation of RsfR with acetyl phosphate resulted in a decrease in the dephosphorylation rate of Ms_RsfB by RsfR. In a separate experiment, we compared the levels of RsfR phosphorylation by acetyl phosphate and RsfSTr (Fig. S2). Acetyl phosphate phosphorylated purified RsfR less efficiently than RsfSTr under the given experimental conditions. Despite acetyl phosphate’s lower efficiency in phosphorylating RsfR than RsfSTr, phosphorylation of RsfR by acetyl phosphate resulted in a greater reduction of RsfR phosphatase activity toward Ms_RsfB than that by RsfSTr. The discrepancy in results might be attributed to variations in the conditions between the two experiments, such as the amount of RsfR used or the molar ratio between RsfR and RsfSTr. Additionally, we cannot rule out the possibility that acetyl phosphate inhibits RsfR phosphatase activity to some extent through competitive inhibition. We performed an additional experiment to reinforce our finding that phosphorylation of the aspartate conserved in the receiver domain of RsfR by RsfS reduces RsfR phosphatase activity. We compared the phosphatase activity of the D74E phosphomimetic form of RsfR with that of the WT form of RsfR. As shown in Figure 6*E*, RsfRD74E almost lost its phosphatase activity toward Ms_RsfB, clearly indicating the phosphorylation-induced reduction in RsfR phosphatase activity.

**Figure 7:**
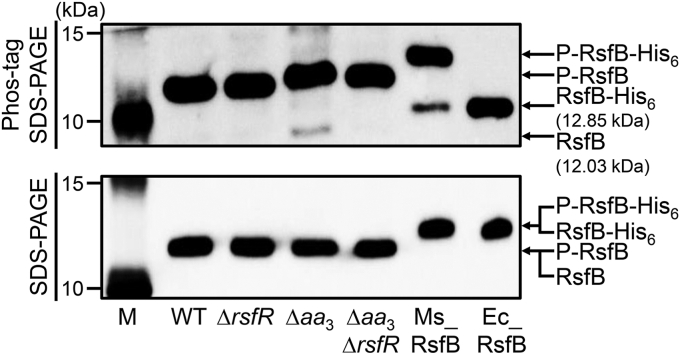
**Phosphorylation levels of RsfB in the WT, Δ*rsfR*, Δ*aa***_**3**_**, and Δ*aa***_**3**_**Δ*rsfR* strains of *Mycobacterium smegmatis*.** The strains were grown aerobically to an *A*_600_ of 0.45 to 0.5 in 7H9-glucose medium. Cell-free crude extracts (10 μg) of the strains were separated on both SDS-PAGE and 50 μM Mn^2+^-Phos-tag SDS-PAGE, followed by Western blotting analysis with RsfB polyclonal antibodies. The bands representing unphosphorylated RsfB (RsfB) and phosphorylated RsfB (P-RsfB) are indicated by the *arrows*. 0.01 μg each of RsfB purified from *Escherichia coli* and *M. smegmatis* were included to indicate unphosphorylated His_6_-tagged RsfB (RsfB-His_6_) and phosphorylated His_6_-tagged RsfB (P-RsfB-His_6_), respectively. M, molecular weight marker lanes.

Using Phos-tag SDS-PAGE and subsequent Western blotting analyses, we comparatively examined the phosphorylation state of RsfB in the WT, Δ*aa*_3_, Δ*rsfR*, and Δ*aa*_3_Δ*rsfR* mutant strains of *M. smegmatis* that were grown aerobically (Fig. 7). His_6_-tagged RsfB purified from *M. smegmatis* and *E. coli* was included in the experiment to indicate the position of phosphorylated and unphosphorylated RsfB on the Phos-tag SDS-PAGE gel, respectively. In the WT and Δ*rsfR* mutant strains of *M. smegmatis*, RsfB was found to be present almost exclusively in a phosphorylated form. Unphosphorylated RsfB, which is an active form of RsfB as an anti-SigF antagonist, appeared in the Δ*aa*_3_ mutant in contrast to the WT and Δ*rsfR* mutant strains, although the major fraction of RsfB still existed in a phosphorylated form. Inactivation of *rsfR* in the Δ*aa*_3_ mutant (Δ*aa*_3_Δ*rsfR*) abolished the appearance of unphosphorylated RsfB observed for the Δ*aa*_3_ mutant, indicating that the increased fraction of unphosphorylated RsfB in the Δ*aa*_3_ mutant is the result of the action of the RsfR phosphatase.

### Expression of *MSMEG_1777* is strongly induced under conditions that inhibit the respiratory ETC

**Figure 8:**
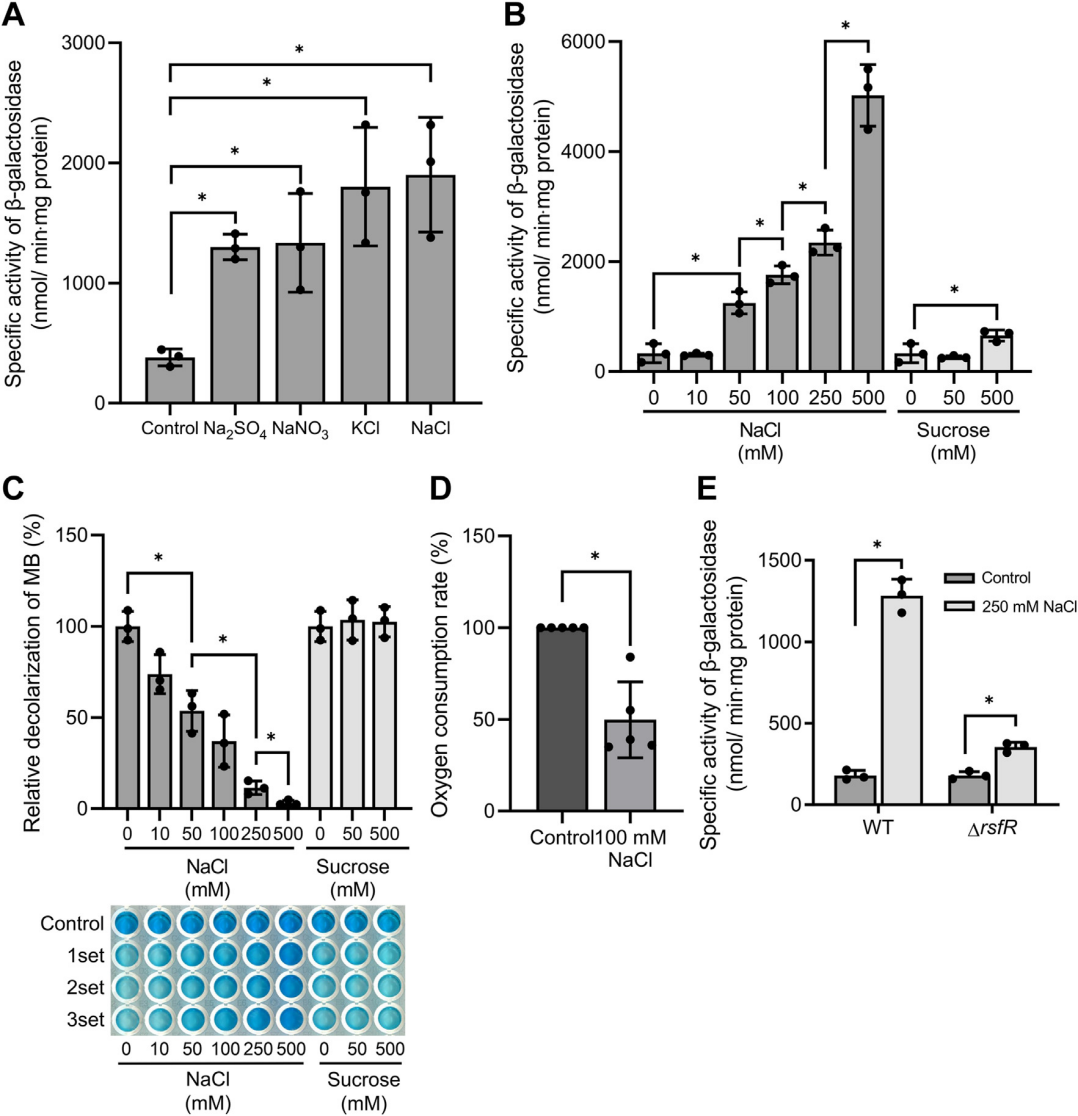
**Effects of an increase in extracellular ionic strength on *MSMEG_1777* expression and the respiration rate of *Mycobacterium smegmatis*.***A*, expression of *MSMEG_1777* in the WT strain of *M. smegmatis* treated with various salts. The *M. smegmatis* strain harboring pNCII1777 was grown aerobically to an *A*_600_ of 0.45 to 0.5 in 7H9-glucose medium and further incubated for 2 h, following the addition of the salts to a final concentration of 50 mM. Expression of *MSMEG_1777* was quantified by determining β-galactosidase activity in the strains. *B*, expression of *MSMEG_1777* in the WT strain treated with increasing concentrations of NaCl or sucrose. The *M. smegmatis* strain carrying pNCII1777 was grown aerobically to an *A*_600_ of 0.45 to 0.5 in 7H9-glucose medium and further incubated for 2 h, following the addition of NaCl or sucrose to the cultures at the indicated concentrations. Expression of *MSMEG_1777* was quantified by determining β-galactosidase activity in the strains. *C*, effect of NaCl or sucrose on the oxygen consumption rate of *M. smegmatis*. The oxygen consumption rate of the strains was extrapolated from the extent of decolorization of MB as described in Experimental procedures. Positive controls included cell suspensions untreated with NaCl (sucrose), while cell-free 7H9-glucose media containing NaCl or sucrose at specified concentrations served as negative controls. The decolorization extent of the negative and positive controls is set at 0 and 100, respectively, and the relative values are expressed for the experimental groups. *D*, the oxygen consumption rates of the NaCl-untreated (control) and NaCl-treated (100 mM) WT strain were also measured using a Clark-type electrode. The oxygen consumption rate of the control is set as 100, and the relative value is expressed for the NaCl-treated strain. *E*, expression of *MSMEG_1777* in the WT and Δ*rsfR* mutant strains treated with NaCl. The *M. smegmatis* strains harboring pNCII1777 were grown aerobically to an *A*_600_ of 0.45 to 0.5 in 7H9-glucose medium, and the cultures were either treated with NaCl to a final concentration of 250 mM for 2 h or untreated with NaCl as controls. Expression of *MSMEG_1777* was quantified by determining β-galactosidase activity in the strains. All values are the means of the results from three biological replicates for *panel A*, *B*, *C*, and *E* and five biological replicates for *panel D*. The error bars indicate the SDs. ∗*p* < 0.05. MB, methylene blue.

It has been suggested that the CHASE3 domain is implicated in the sensing of salt stress (32, 33). Due to the presence of the CHASE3 domain in the N-terminal sensory domain of the RsfS HK, we examined whether expression of *MSMEG_1777* in *M. smegmatis* is changed in response to salt stress. As shown in Figure 8*A*, when aerobic cultures of *M. smegmatis* were treated with various salts (NaCl, KCl, NaNO_3_, and Na_2_SO_4_), the expression level of *MSMEG_1777* was significantly increased regardless of the sorts of cations and anions constituting the salts, indicating that a specific ion or salt is not responsible for activation of the SigF transcriptional activity. We next examined whether induction of *MSMEG_1777* expression by salt treatment was a consequence of increased osmolarity or ionic strength. Increasing concentrations of NaCl in growth medium are expected to lead to a rise in both ionic strength and osmolarity, while increasing concentrations of nonionic sucrose raise the osmolarity of growth medium without affecting ionic strength. As shown in Figure 8*B*, the expression level of *MSMEG_1777* in the WT strain of *M. smegmatis* was gradually increased with increasing concentrations of treated NaCl. In contrast, treatment of the *M. smegmatis* culture with 50 mM sucrose did not cause the increased expression of *MSMEG_1777*, and even treatment of 500 mM sucrose, which is equivalent to 250 mM NaCl in osmolarity, resulted in only a 2-fold increase in *MSMEG_1777* expression. This result implies that the activation of SigF functionality by salt treatment is not brought about by osmotic stress.

Based on the previous report that respiration of *E. coli* is inhibited by treatment of high concentrations of NaCl (34) together with our finding that inhibition of the respiratory ETC leads to a strong induction of the SigF regulon (22), we assumed that it might be respiration inhibition that causes the increased expression of *MSMEG_1777* in *M. smegmatis* exposed to salt stress. Using methylene blue (MB), an indicator of dissolved oxygen levels in an aqueous solution, we examined whether an increase in the NaCl concentration in growth medium inhibits the respiration of *M. smegmatis*. As shown in Figure 8*C*, the respiration rate of *M. smegmatis* was decreased with increasing concentrations of NaCl added to growth medium, whereas there was no change in the respiration rate of *M. smegmatis* when the culture was treated with sucrose to a final concentration of 50 mM or 500 mM. To confirm the observed respiration inhibition by NaCl treatment, we also determined the effect of NaCl treatment on the oxygen consumption rate of *M. smegmatis* using a Clark-type electrode. As shown in Figure 8*D*, *M. smegmatis* treated with 100 mM NaCl showed a decrease in the oxygen consumption rate by about 50% compared to the untreated control strain. Taken together, these results suggest that induction of *MSMEG_1777* expression under high ionic strength conditions might be a result of respiration inhibition. To examine whether the RsfSR TCS is required for the increased expression of *MSMEG_1777* under high ionic strength conditions, we determined the expression level of *MSMEG_1777* in the WT and Δ*rsfR* mutant strains grown under NaCl-treated and NaCl-untreated conditions. Treatment of the aerobic cultures with 250 mM NaCl led to a 7-fold increase in *MSMEG_1777* expression in the WT strain relative to the NaCl-untreated control, whereas *MSMEG_1777* expression in the Δ*rsfR* strain was only 2-fold induced by NaCl treatment (Fig. 8*E*). This result suggests that the RsfSR TCS plays an important role in an increase in SigF functionality in *M. smegmatis* exposed to high ionic strength conditions and that there is a factor other than the RsfSR TCS that might contribute to a slight enhancement of SigF functionality under the conditions.

**Figure 9:**
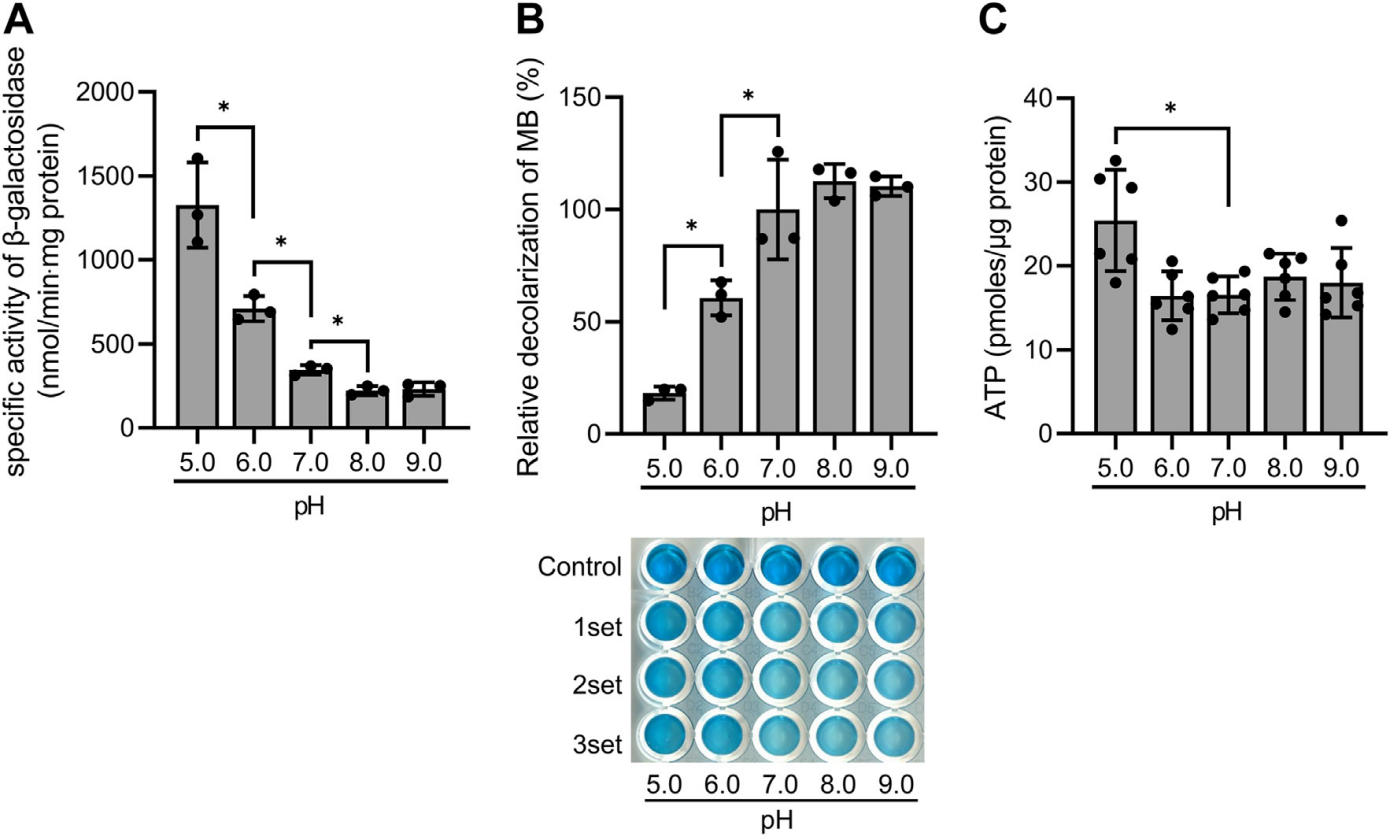
**Effects of changes in extracellular pH on *MSMEG_1777* expression, intracellular ATP levels, and the respiration rate of *Mycobacterium smegmatis*.***A*, expression of *MSMEG_1777*. *B*, the respiration rate. *C*, intracellular levels of ATP. The WT strain of *M. smegmatis* harboring pNCII1777 was grown aerobically to an *A*_600_ of 0.45 to 0.5 in 7H9-glucose medium, whose pH was adjusted to pH 5, 6, 7, 8, or 9. Cell crude extracts were used for β-galactosidase assay to determine the expression rate of *MSMEG_1777*. The oxygen consumption rate of the WT strain of *M. smegmatis* was extrapolated from the decolorization rate of MB as described in Experimental procedures. The positive control involved a cell suspension in 7H9-glucose+Tween 80 medium at pH 7, while the cell-free 7H9-glucose media with pH adjusted to 5, 6, 7, 8, or 9 served as negative controls. The decolorization extent of the negative and positive controls is set at 0 and 100, respectively, and the relative values are expressed concerning those of the experimental groups. ATP levels were determined using the WT strain of *M. smegmatis* grown aerobically to an *A*_600_ of 0.45 to 0.5 under indicated pH conditions. The error bars indicate the SDs. All values are the means of the results from three biological replicates for *panel A* and *B* and six biological replicates for *panel C*. The error bars indicate the SDs. ∗*p* < 0.05. MB, methylene blue.

Changes in the extracellular pH are expected to affect the proton motive force across the membrane and thus alter the functionality of the respiratory ETC and intracellular levels of ATP. By measuring the expression level of *MSMEG_1777* in the WT strain of *M. smegmatis* grown under different pH conditions, we investigated the correlation between changes in the respiration rate, the intracellular ATP concentration, and SigF functionality. As shown in Figure 9*A*, the expression level of *MSMEG_1777* was increased with decreasing pH of growth medium in the pH range of 5 to 8. In the same pH range, the lower the extracellular pH values, the lower the respiration rate of *M. smegmatis* as judged by decolorization of MB (Fig. 9*B*). The intracellular concentrations of ATP in *M. smegmatis* grown at different pH conditions ranging from pH 6 to pH 9 were almost constant, but the ATP concentration in *M. smegmatis* grown at pH 5 was higher by 47% than that in the strain grown at pH 7 (Fig. 9*C*), which is consistent with the previous report that the intracellular level of ATP is increased in *E. coli* and *Pseudomonas aeruginosa* under low pH conditions (35, 36). Taken together, these results imply that the increased expression of *MSMEG_1777* at low pH is not due to a decrease in the intracellular ATP concentration but possibly the result of inhibition of the respiratory ETC.

**Figure 10:**
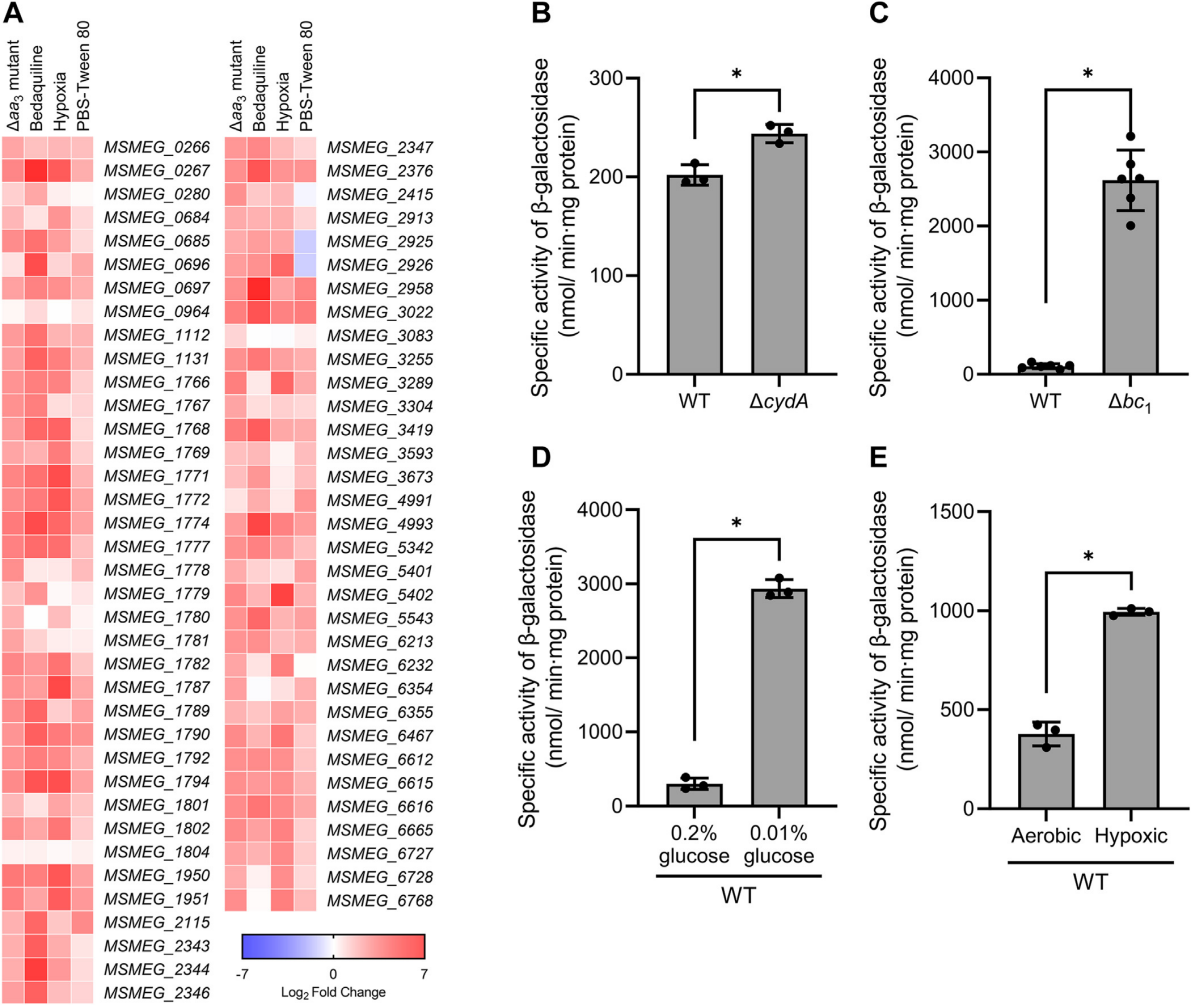
**Transcriptional profiles of the SigF regulon in *Mycobacterium smegmatis* under respiration-inhibitory conditions.***A*, heatmap. The heatmap shows the relative expression of the SigF regulon in the WT strain of *M. smegmatis* exposed to various stress conditions [treatment of bedaquiline, hypoxia, and starvation (PBS-Tween 80)] that are expected to inhibit the respiratory ETC, compared to the WT strain grown aerobically without exposure to the stress conditions (control). The expression of the SigF regulon in the Δ*aa*_3_ mutant of *M. smegmatis* relative to the WT strain is also included in the heatmap. The transcriptomic data used in the generation of the heatmap were retrieved from NCBI’s Gene Expression Omnibus using the following accession number: Δ*aa*_3_ mutant (GSE155251), a *sigF* mutant (GSE19774), hypoxia (GSE128412), PBS-Tween 80 (GSE69983), bedaquiline (GSE59871). The *color and shading* of each cell in the heatmap denote the log_2_ fold change in gene expression (log_2_FC) in the experimental groups *versus* the control group. The WT and Δ*aa*_3_ mutant strains of *M. smegmatis* were grown to an *A*_600_ of 0.45 to 0.5 in 7H9-glucose medium (40). The transcriptomic data were obtained from the WT strain of *M. smegmatis* exposed to 2 mg/L of bedaquiline for 60 min (39), hypoxic conditions for 24 h (37), or PBS-Tween 80 medium for 60 min (38). The downregulated genes with log_2_FC < −4 and *p* value <0.05 in a *sigF* mutant strain relative to the WT strain were selected as the genes belonging to the SigF regulon (75). *B* and *C*, expression of *MSMEG_1777* in the WT, Δ*cydA*, and Δ*bc*_1_ strains of *M. smegmatis*. The *M. smegmatis* strains harboring pNCII1777 were grown aerobically to an *A*_600_ of 0.45 to 0.5 in 7H9-glucose medium. *D*, expression of *MSMEG_1777* in the WT strain of *M. smegmatis* grown under glucose-replete or glucose-limiting conditions. The *M. smegmatis* strain harboring pNCII1777 was grown aerobically to an *A*_600_ of 0.45 to 0.5 in 7H9 medium supplemented with 0.2 or 0.01% (w/v) glucose. *E*, expression of *MSMEG_1777* in the WT strain of *M. smegmatis* grown under aerobic or hypoxic conditions. The WT strain harboring pNCII1777 was grown aerobically to an *A*_600_ of 0.7 to 0.75 in 7H9-glucose medium. The cultures were immediately harvested (aerobic) or subjected to further hypoxic incubation for 3 h (hypoxic). Expression of *MSMEG_1777* was quantified by determining β-galactosidase activity in the strains. All values are the means of the results from three biological replicates for *panel B*, *D*, and *E* and six biological replicates for *panel C*. The error bars indicate the SDs. ∗*p* < 0.05. ETC, electron transport chain.

By compiling several deposited transcriptomic data obtained from *M. smegmatis* exposed to conditions likely to inhibit the respiratory ETC such as hypoxia, starvation (PBS-Tween 80), and bedaquiline (an inhibitor of ATP synthase) treatment (37, 38, 39), we found that expression of the SigF regulon is significantly increased under the respiration-inhibitory conditions as in the Δ*aa*_3_ mutant (Fig. 10*A*) (40). To confirm that inhibition of the respiratory ETC leads to induction of the SigF regulon, we determined the expression level of *MSMEG_1777* in ETC mutants of *M. smegmatis* other than the Δ*aa*_3_ strain. The Δ*cydA* strain is a mutant in which the *bd* quinol oxidase, the minor terminal oxidase in the ETC of *M. smegmatis*, is inactivated, and the Δ*bc*_1_ strain is a mutant of the cytochrome *bcc*_1_ complex that constitutes the main branch of the respiratory ETC with the *aa*_3_ cytochrome *c* oxidase. When the strains were grown aerobically, the Δ*bc*_1_ mutant showed a significantly higher expression of *MSMEG_1777* than the WT strain, while only a marginal increase in *MSMEG_1777* expression was observed for the Δ*cydA* mutant relative to the WT strain (Fig. 10, *B* and *C*). When the WT strain of *M. smegmatis* was exposed to glucose-limiting (0.01% glucose) or hypoxic conditions, expression of *MSMEG_1777* was significantly upregulated (Fig. 10, *D* and *E*), confirming the results of Figure 10*A*.

## Discussion

The mycobacterial SigF is homologous to the general stress response sigma factor SigB found in *Firmicutes* such as *Bacillus subtilis*, *Bacillus cereus*, and *Listeria monocytogenes*. The functionality of the SigF homologs is controlled by the PSS composed of their cognate antisigma factors and antisigma factor antagonists (Fig. 1). Among the PSS components, the antisigma factor antagonist serves as the major control point that regulates the functionality of the PSS by reflecting environmental signals. The antisigma factor antagonist alters its functionality either by recognizing environmental changes by itself (i.g., RsfA) (11, 21, 22) or through covalent modification such as phosphorylation by (an) upstream regulatory module(s) that recognize(s) environmental signals. For example, RsbV, an anti-SigB antagonist in *B. subtilis*, is phosphorylated by RsbW and dephosphorylated to become activated by two PP2C-type phosphatases, RsbP and RsbU (41, 42, 43). The RsbQ α/β hydrolase-like protein and RsbP phosphatase are required to increase SigB functionality in response to a decrease in intracellular ATP levels (44, 45). On the other hand, the 25S stressosome complex containing RsbT, RsbS, and RsbR is involved in SigB activation under environmental stress conditions such as osmotic stress, heat shock, and low pH (42, 46, 47, 48, 49, 50). Under these conditions, RsbT phosphorylates RsbS with the help of RsfR in the stressosome complex and dissociates from the RsfR–RsfS complex, thereby interacting with the N-terminal domain of RsbU to activate the phosphatase activity of RsbU (46). In the case of *B. cereus*, the functionality of SigB was suggested to be regulated by the SigB PSS combined with the RsbKY TCS as a sensing module in a way similar to SigF in *M. smegmatis* (49, 51, 52, 53). The RsbK HK is a hybrid-type HK with a receiver domain at its C terminus, and RsbY is a receiver domain–containing PP2C-type phosphatase that dephosphorylates the anti-SigB antagonist RsbV (51, 54). The RsbK HK is required for SigB activation under heat shock and osmotic stress conditions. The cGMP-specific phosphodiesterases, adenylyl cyclases and FhlA (GAF) and CHASE3 domains in RsbK were predicted to function as sensor domains that recognize intracellular and extracellular signals, respectively, but the mechanism underlying the recognition of the stress signals remains unsolved. Under these stress conditions, the RsbK HK phosphorylates the receiver domain of RsbY directly or *via* histidine phosphotransferase to activate RsbY phosphatase activity (51, 55). It was also suggested that the methyltransferase RsbM is involved in the regulation of the RsbK kinase activity by methylation of RsbK (56, 57).

**Figure 11:**
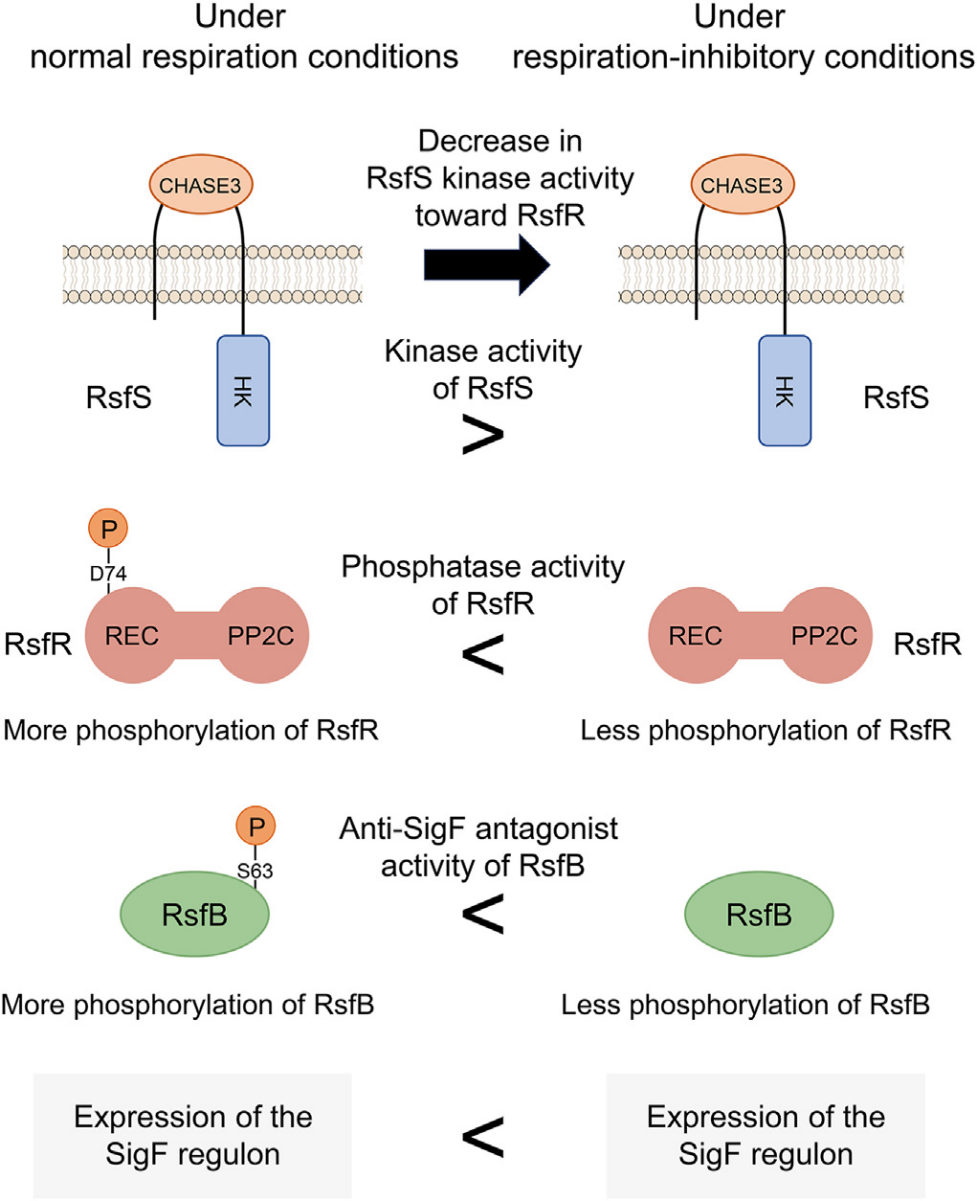
**Model for the regulation of RsfSR TCS and RsfB functionality in response to respiration inhibition in *Mycobacterium smegmatis.*** Under respiration-inhibitory conditions, the kinase activity of RsfS toward RsfR in *M. smegmatis* is likely to decrease, resulting in an increase in the intracellular fraction of unphosphorylated RsfR. Since unphosphorylated RsfR has greater phosphatase activity on phosphorylated RsfB than phosphorylated RsfR, the fraction of the active unphosphorylated RsfB is increased under respiration-inhibitory conditions, which leads to upregulation of the SigF regulon. TCS, two-component system.

In *M. smegmatis* and *M. tuberculosis*, the sensory module that recognizes environmental signals and regulates the functionality of the SigF PSS has remained to be elusive. Based on the genetic organization of the *rsfB* locus and their relatedness to the RsbKY TCS of *B. cereus*, we presumed that the *rsfS* and *rsfR* gene products participate in the regulation of the phosphorylation state of RsfB. We found that an increase in both *MSMEG_1777* expression and the cellular abundance of the dephosphorylated active form of RsfB observed for the Δ*aa*_3_ mutant relative to the WT strain is abolished by null mutation of *rsfR* (Figs. 2*B* and 7). We also found that RsfS-mediated phosphorylation of RsfR reduces the phosphatase activity of RsfR toward phosphorylated RsfB (Fig. 6). Taken together, these findings suggest the following model (Fig. 11). (i) The kinase activity of RsfS is likely to be reduced under respiration-inhibitory conditions, resulting in the increased phosphatase activity of RsfR toward phosphorylated RsfB due to less phosphorylation of RsfR. (ii) Increased dephosphorylation of RsfB by the enhanced RsfR phosphatase activity is responsible for induction of the SigF regulon under respiration-inhibitory conditions.

The RsfSR TCS of *M. smegmatis* is similar to the RsbKY TCS of *B. cereus* in that they act as the sensing modules regulating the phosphorylation state of the antisigma factor antagonists and are composed of a CHASE3 domain–containing HK and a PP2C-type phosphatase. However, they have several differences as follows, which implies differences in signal sensing and regulatory mechanism between RsfSR and RsbKY (51, 55, 56). (i) Phosphorylation of RsbY by the RsbK HK increases RsbY phosphatase activity in *B. cereus*, whereas phosphorylation of RsfR by the RsfS HK decreases RsfR phosphatase activity in *M. smegmatis*. (ii) While the RsbK HK of *B. cereus* possesses two putative sensing domains, CHASE3 and GAF, RsfS has only the CHASE3 domain. (iii) The RsbM methyltransferase inhibits phosphotransfer from RsbK to RsbY by methylation of RsbK under SigB-nonactivating conditions in *B. cereus*. In contrast, there is no RsbM homolog in *M. smegmatis*. (iv) While RsbK of *B. cereus* is a hybrid-type sensor HK containing a receiver domain, RsfS of *M. smegmatis* is a prototypical HK.

The CHASE3 domain is a putative extracellular sensory domain found in sensory proteins such as sensor HKs, methyl-accepting chemoreceptor proteins, adenylate cyclases, and diguanylate cyclases/phosphodiesterases (58, 59, 60). The domain consists of 130 to 150 amino acids and is often found in combination with other sensory domains such as Per-Arnt-Sim and GAF in the sensory proteins (58, 59, 60). The previous studies on the CHASE3 domain–containing HKs, CfcA of *Pseudomonas putida* and KipF of *Sphingomonas melonis*, have suggested that the domain is involved in salt (high ionic strength) sensing like the RsfS HK of *M. smegmatis* (32, 33). However, it remains obscure how the CHASE3 domains in CfcA and KipF are involved in recognition of salt stress. A clue regarding how the CHASE3 domain recognizes the condition of high ionic strength came from the following finding. Seemingly unrelated environmental conditions such as low pH, high ionic strength, depletion of the energy source, and hypoxia serve as triggers to induce the genes transcribed by SigF (Figure 8, Figure 9, Figure 10). Given that a common effect of the above environmental conditions on *M. smegmatis* cells is inhibition of the respiratory ETC, we can assume that the CHASE3 domain might be a sensory domain that can sense the functional state of the respiratory ETC. This assumption is in line with the RsfSR-dependent induction of expression of the SigF regulon under conditions of respiration inhibition such as inactivation of the *aa*_3_ cytochrome *c* oxidase or cytochrome *bcc*_1_ complex by mutation (Fig. 10*C*) (22). If it were true that inhibition of the respiratory ETC causes induction of the SigF regulon, the next question is what serves as a signal to directly activate the SigF PSS during inhibition of the respiratory ETC in *M. smegmatis*. Given that inhibition of the respiratory ETC causes a decrease in the formation of the proton motive force across the cytoplasmic membrane, it is possible that a decrease in intracellular levels of ATP might be the signal that activates the SigF PSS as in the SigB PSS of *B. subtilis* (44, 61). Alternatively, the reduced proton gradient across the membrane itself or the entailed changes in the membrane potential or the redox state of an electron carrier such as menaquinone/menaquinol might serve as a signal to activate the SigF PSS. We demonstrated that expression of *MSMEG_1777* is significantly induced in *M. smegmatis* grown at pH 5 compared to the control strain grown at pH 7 despite an increase in both intracellular levels of ATP and proton gradient across the cytoplasmic membrane in *M. smegmatis* grown at pH 5 relative to the control strain (Fig. 9). This finding indicates that the major signal to activate the SigF PSS by inhibition of the respiratory ETC is neither a reduction in ATP levels nor collapse of the proton motive force. However, we cannot rule out the possibility that a decrease in intracellular ATP levels under respiration-inhibitory conditions makes the anti-SigF antagonist RsfB somewhat less phosphorylated and thus slightly activates the SigF PSS since ATP is required for the kinase activity of RsbW2 which phosphorylates RsfB. This possibility might explain our finding that expression of *MSMEG_1777* is marginally increased in an RsfSR-independent way by inactivation of the *aa*_3_ oxidase (Figs. 2*B* and 3A). Currently, we do not know how the RsfS HK recognizes inhibition of the respiratory ETC and what role the CHASE3 domain plays in signal recognition. Further study is necessary to answer these unsolved questions.

In conclusion, respiration-inhibitory conditions refer to a broad range of conditions in which the functionality of the respiratory ETC is reduced. They include conditions in which electron donors or terminal electron acceptors of the ETC are deficient (nutrient starvation and hypoxia), conditions in which components of the respiratory ETC are inhibited (ETC mutants and treatment of ETC inhibitors), and conditions in which the proton motive force and membrane potential are affected so that the ETC is inhibited (low pH, high ionic strength, and inhibition of the F_o_/F_1_ ATP synthase). In this study, we found that the transcriptional activity of SigF is activated in *M. smegmatis* under conditions inhibiting the respiratory ETC, which implies that SigF plays an important role in sensing and integrating the environmental and intracellular signals that affect the respiratory ETC to regulate gene expression. On top of our previous report that had revealed the PSS regulating SigF functionality (22), we here identified the RsfSR TCS that regulates the functionality of the SigF PSS at the control point of the anti-SigF antagonist RsfB in response to change in the functional state of the respiratory ETC. We also suggest that the CHASE3 domain might serve as a sensory domain that recognizes the state of the respiratory ETC.

## Experimental procedures

### Bacterial strains, plasmids, and culture conditions

The bacterial strains and plasmids used in this study are listed in Table S1. *E. coli* strains were cultivated in LB medium on a gyratory shaker (200 rpm) at 37 °C. *M. smegmatis* strains were grown aerobically in Middlebrook 7H9 medium (Difco) supplemented with 0.2% (w/v) glucose (7H9-glucose) and 0.02% (v/v) Tween 80 as an anticlumping agent on a gyratory shaker at 37 °C. For glucose-limiting conditions, *M. smegmatis* strains were grown aerobically in 7H9 medium supplemented with 0.01% (w/v) glucose and 0.02% (v/v) Tween 80. *M. smegmatis* strains were grown under hypoxic conditions in an 100 ml flask filled with 80 ml of aerobically grown culture with an absorbance at 600 nm (*A*_6__00_) of 0.7 to 0.75 and tightly sealed with a rubber stopper on a gyratory shaker (200 rpm) at 37 °C for 3 h, which allowed a gradual depletion of O_2_ from the growth medium. Ampicillin (100 or 200 μg/ml for *E. coli*), kanamycin (50 μg/ml for *E. coli* and 15 or 30 μg/ml for *M. smegmatis*), and hygromycin (200 μg/ml for *E. coli* and 50 μg/ml for *M. smegmatis*) were added to growth medium when required. For treatment of *M. smegmatis* cultures with NaCl, KCl, Na_2_SO_4_, NaNO_3_, and sucrose, the cultures were grown to an *A*_600_ of 0.45 to 0.5 and further incubated for 2 h, following the addition of the salts or sucrose to the cultures at the indicated concentrations. The construction of the mutants and plasmids used in this study is described in Supporting information.

### DNA manipulation and transformation

Standard protocols and manufacturers’ instructions were followed for recombinant DNA manipulations (62). Transformation of *M. smegmatis* with plasmids was conducted by electroporation as previously described (63).

### Site-directed mutagenesis

To introduce point mutations into the *rsfR* gene, PCR-based mutagenesis was performed using the Quick Change site-directed mutagenesis procedure (Stratagene). Synthetic oligonucleotides containing a mutated codon in the middle of their sequences were used to mutagenize the original codons. The primers used for mutagenesis are listed in Table S2. Mutations were verified by DNA sequencing.

### β-Galactosidase assay and determination of the protein concentration

Cells of *M. smegmatis* were harvested, resuspended in β-galactosidase assay buffer [50 mM potassium phosphate buffer (pH 7), 10 mM KCl, 1 mM MgSO_4_, and 20 mM β-mercaptoethanol], and broken by sonication using a VCX-750 sonicator (Sonic and Materials, Inc). Cell-free crude extracts were obtained following centrifugation at 20,000*g* for 10 min at 4 °C. The β-galactosidase activity was assayed spectrophotometrically following the procedure described elsewhere (64). The protein concentration was determined using a Bio-Rad protein assay kit (Bio-Rad) with bovine serum albumin as a standard protein.

### Protein purification

#### RsfR

The C terminally His_6_-tagged WT and mutant forms of RsfR were expressed in *E. coli* BL21 (DE3) strains harboring the pT7-7 derivative plasmids (pT7-7rsfR, pT7-7rsfRD74A, and pT7-7rsfRD74E). The strains were cultivated aerobically at 37 °C in LB medium containing 100 μg/ml ampicillin to an *A*_600_ of 0.4 to 0.6. Expression of the WT and mutant forms of *rsfR* was induced by the addition of IPTG to a final concentration of 0.5 mM and then cells were further grown for 4 h at 30 °C. After 400 ml of *E. coli* cultures were harvested, cells were resuspended in 10 ml of buffer A [20 mM Tris–HCl (pH 8), 100 mM NaCl] containing 10 U/ml DNase I and 10 mM MgCl_2_. The resuspended cells were disrupted twice using a French press (Thermo Fisher Scientific), and cell-free crude extracts were obtained by centrifugation twice at 27,000*g* for 15 min at 4 °C. 300 μl of 80% (v/v) slurry (bed volume: 240 μl) of Ni-Sepharose high performance resin (GE Healthcare) was packed into a column. After equilibration of the resin with ten bed volumes of buffer A, cell-free crude extracts were loaded into the column. The resin was washed with 40 bed volumes of buffer A containing 10 mM imidazole, 20 bed volumes of buffer A containing 30 mM imidazole, and then His_6_-tagged RsfR was eluted with ten bed volumes of buffer A containing 100 mM imidazole. The eluted His_6_-tagged RsfR was diluted by 10-fold with buffer A and subjected to affinity chromatography again to enhance the purity of RsfR.

#### N terminally RsfSTr

Overexpression of C terminally His_6_-tagged RsfSTr (amino acids 190–525) was conducted using *E. coli* BL21 carrying pETrsfSTr in the same way as RsfR overexpression. After 800 ml of the *E. coli* culture was harvested, cells were resuspended in 30 ml of buffer A containing 10 U/ml DNase I, 10 mM MgCl_2_, 20 mM β-mercaptoethanol, and 1 mM PMSF and disrupted by French pressure. After centrifugation, cell-free crude extracts were loaded into a column packed with Ni-Sepharose resin. The resin was washed with 40 bed volumes of buffer A containing 10 mM imidazole and 20 mM β-mercaptoethanol, 20 bed volumes of buffer A containing 50 mM imidazole and 20 mM β-mercaptoethanol, and then His_6_-tagged RsfSTr was eluted with 7.5 bed volumes of buffer A containing 250 mM imidazole and 20 mM β-mercaptoethanol.

#### MSMEG_6128

Overexpression of C terminally His_6_-tagged MSMEG_6128 was conducted using *E. coli* BL21 carrying pT7-7MSMEG6128 in the same way as RsfR overexpression. After 400 ml of *E. coli* cultures were harvested, cells were resuspended in 10 ml of buffer A containing 10 U/ml DNase I and 10 mM MgCl_2_. The resuspended cells were disrupted twice using a French press. After centrifugation, cell-free crude extracts were loaded into a column packed with Ni-Sepharose resin. The resin was washed with 40 bed volumes of buffer A containing 10 mM imidazole, 20 bed volumes of buffer A containing 50 mM imidazole, and then His_6_-tagged MSMEG_6128 was eluted with ten bed volumes of buffer A containing 250 mM imidazole.

#### RsfB

Puriﬁcation of C terminally His_6_-tagged RsfB from *M. smegmatis* and *E. coli* was conducted as described previously (22).

All purified proteins were subjected to gel-filtration chromatography using a PD-10 desalting column (GE Healthcare) to remove NaCl and imidazole.

### Determination of protein phosphorylation using Phos-tag SDS-PAGE

#### Dephosphorylation of RsfB by RsfR

The dephosphorylation reaction was started by the addition of phosphorylated RsfB purified from *M. smegmatis* to the reaction mixture [300 mM Tris-HCl (pH 8), 50 mM KCl, 10 mM MgCl_2_, and 1 mM ATP] containing purified RsfR and continued for the appropriate time at 30 °C. To examine the effect of RsfR phosphorylation on RsfR phosphatase activity toward phosphorylated RsfB, RsfR was phosphorylated by RsfSTr or acetyl phosphate. For phosphorylation of RsfR with RsfSTr, purified RsfR was mixed with RsfSTr in the reaction mixture described above and the reaction proceeded for 30 min at 30 °C. Alternatively, acetyl phosphate was added to the reaction mixture containing purified RsfR to a final concentration of 40 mM, and the reaction proceeded for 30 min at 30 °C for RsfSTr-independent phosphorylation of RsfR. Subsequently, phosphorylated RsfB was added to the reaction mixtures containing phosphorylated RsfR, and the reactions continued for the appropriate time at 30 °C. The reactions were terminated by adding 3x gel-loading buffer [195 mM Tris-HCl (pH 6.8), 30% (w/v) glycerol, 3% (w/v) SDS, 15% (v/v) β-mercaptoethanol, and 0.1% (w/v) bromophenol blue]. Dephosphorylation of RsfB was detected by means of Phos-tag SDS-PAGE using 12.5% (w/w) acrylamide gel containing 50 μM Mn^2+^-Phos-tag (50 μM Phos-tag acrylamide and 100 μM MnCl_2_) prepared as described elsewhere (65). The gels were stained with Coomassie brilliant blue.

#### Phosphorylation of RsfR by RsfS

Autophosphorylation of RsfSTr was performed in the reaction mixture [300 mM Tris-HCl (pH 8), 50 mM KCl, 10 mM MgCl_2_, and 1 mM ATP] for 30 min at 30 °C. After the autophosphorylation reaction, the purified WT and mutant forms of RsfR were added to the autophosphorylation reaction mixtures for the phosphotransfer reaction. The reactions were performed at 30 °C and terminated at various time intervals by adding 3x gel-loading buffer. The samples were not boiled prior to electrophoresis and instead kept on ice for more than 30 min to prevent hydrolysis of phospho-Asp. Phosphorylation of RsfR was determined by Phos-tag gels composed of 8% (w/w) acrylamide gel containing 75 μM Mn^2+^-Phos-tag (75 μM Phos-tag acrylamide and 150 μM MnCl_2_), which is optimized to detect phosphorylated RsfR. The gels were stained with Coomassie brilliant blue.

### Western blotting analysis

To detect His_6_- and 2B8 epitope-tagged proteins as well as untagged GroEL and RsfB in cells, Western blotting analysis was performed as described previously (65, 66). Cell-free crude extracts were subjected to SDS-PAGE or Phos-tag SDS-PAGE, and proteins on gels were transferred to polyvinylidene ﬂuoride membranes (Millipore). SDS-PAGE gels were equilibrated in transfer buffer [20% (v/v) methanol, 48 mM Tris, 39 mM glycine, 1.3 mM SDS (pH 9.2)]. Phos-tag SDS-PAGE gels were pretreated with transfer buffer containing 10 mM EDTA for 10 min to remove Mn^2+^ from the gels, followed by incubation for 10 min in transfer buffer without EDTA. Transfer of proteins from the gels to polyvinylidene ﬂuoride was performed using a semidry transfer apparatus (Bio-Rad) at a constant voltage of 20 V for 30 min. To detect 2B8-tagged proteins, a mouse monoclonal IgG against 2B8 (Biojane, Pyeongtaek-si) was used at a dilution of 1:20,000. To detect His_6_-tagged proteins, a mouse monoclonal IgG against His_6_ (Thermo Fisher Scientific; MA1-21315) was used at a dilution of 1:2000. To detect GroEL, a mouse mAb against Hsp65 (Santa Cruz Biotechnology; sc58170) was employed at a 1:2000 dilution. To detect expressed RsfB in cells, a rabbit polyclonal antibody against the RsfB protein was used at a 1:20,000 dilution. Horseradish peroxidase–conjugated anti-mouse and anti-rabbit IgGs (Bio-Rad) were used at a 1:10,000 and 1:3000 dilution, respectively, for the detection of the primary antibodies. The enhanced chemiluminescence kit (Advansta) was used to visualize protein bands *via* a ChemiDoc imaging system (Bio-Rad).

### Determination of the oxygen consumption rate of *M. smegmatis* cells

#### Oxygen consumption assay using MB

The oxygen consumption rate was measured by using MB as previously described (67) with modifications. The WT strain of *M. smegmatis* was grown aerobically to an *A*_600_ of 0.45 to 0.5 in 7H9-glucose medium. The harvested cells were washed twice with glucose-free 7H9 medium containing 0.02% (v/v) Tween 80 (7H9+Tween 80) and resuspended in ice-chilled 7H9-glucose medium with 0.02% (v/v) Tween 80 (7H9-glucose+Tween 80) to an *A*_600_ of 0.5. To determine the effect of NaCl or sucrose on the oxygen consumption rate, the resuspended cells were treated with NaCl or sucrose at specified concentrations and placed into 96-well plates on ice. The NaCl (sucrose)-untreated cell suspension and cell-free 7H9-glucose+Tween 80 medium containing NaCl or sucrose served as positive and negative controls, respectively. To determine the effect of pH on the oxygen consumption rate, the harvested *M. smegmatis* cells were resuspended in ice-chilled 7H9-glucose+Tween 80 medium whose pH was adjusted to 5, 6, 7, 8, or 9. The cell suspensions adjusted to an *A*_600_ of 0.5 were placed into 96-well plates on ice. The cell suspension at pH 7 served as a positive control, while the cell-free 7H9-glucose+Tween 80 media adjusted to pH 5, 6, 7, 8, or 9 served as a negative control. MB was added to the cell suspensions to a final concentration of 5 or 10 mM. After the addition of 100 μl of mineral oil to the well surface to prevent oxygen diffusion, the absorbance of the plate wells was measured at 665 nm for 1 h at 37 °C using the Multiskan SkyHigh Spectrophotometer (Thermo Fisher Scientific). The oxygen consumption rate of the strains was extrapolated from the extent of decolorization of MB. The decolorization extent of the negative and positive controls is set at 0 and 100, respectively, and the relative values are expressed for the experimental groups.

#### Oxygen consumption assay using a Clark-type electrode

The WT strain of *M. smegmatis* was grown aerobically to an *A*_600_ of 0.45 to 0.5 in 7H9-glucose medium. The harvested cells were washed twice with glucose-free 3-(*N*-morpholino)propanesulfonic acid (MOPS) minimal medium [25 mM MOPS (pH 7.2), 25 mM KCl, 10 mM Na_2_SO_4_, 20 mM NH_4_Cl, 10 mM K_2_HPO_4_, 10 μM FeCl_3_, 2 mM MgSO_4_, and 100 μM CaCl_2_] supplemented with 0.02% (v/v) Tyloxapol (MOPS+Tyloxapol) and resuspended in the same ice-chilled medium to an *A*_600_ of 0.5. To determine the effect of NaCl on the oxygen consumption rate, NaCl was added to the resuspended cells to a final concentration of 100 mM. The oxygen consumption rate was measured polarographically with a Clark-type electrode (YSI Inc) using 5 ml of the resuspended cells. Following equilibration of the cell suspension in the electrode chamber for 12 min at 30 °C, the glucose-dependent oxygen consumption was measured for 30 min at 30 °C after the addition of glucose to a final concentration of 0.2% (w/v).

### Quantitation of cellular ATP levels

Total cellular ATP levels were determined as previously described (68) with some modifications. The WT strain of *M. smegmatis* was cultivated aerobically to an *A*_600_ of 0.45 to 0.5 at 37 °C in 7H9-glucose medium whose pH was adjusted to pH 5, 6, 7, 8, or 9. The cultures were harvested by centrifugation and resuspended in an equal volume of Tris-EDTA buffer [100 mM Tris (pH 7.75), 4 mM EDTA]. Cells were broken three times using a Fastprep-24 bead beater (MP Biomedicals). Cell-free crude extracts were obtained by centrifugation at 19,000*g*, for 5 min at 4 °C, and their protein concentration was determined. The quantitation of ATP levels in the crude extracts was carried out using an ATP bioluminescence assay kit HSII (Roche) following the manufacturer’s instruction. Briefly, the crude extracts were heated at 100 °C for 5 min and cooled on ice for 2 min, followed by centrifugation at 19,000*g* for 5 min at 4 °C to remove denatured proteins. Fifty microliters of the prepared samples or ATP standard solutions were loaded into a black microtiter plate, and the reaction was started by adding 50 μl of luciferase reagent to the samples. Bioluminescence was measured for 1 to 10 s after a 1 s delay using a Mithras LB 940 luminometer (Bertholdy). The levels of ATP in the samples were calculated from the standard curve generated using the ATP standard solutions and normalized to the protein concentration of the samples.

## Data availability

The transcriptomic data used in Figure 10*A* were obtained from the NCBI GEO database using the following accession number: Δ*aa*_3_ mutant (GSE155251), a *sigF* mutant (GSE19774), hypoxia (GSE128412), PBS-Tween 80 (GSE69983), bedaquiline (GSE59871).

## Supporting information

This article contains supporting information (22, 31, 63, 69, 70, 71, 72, 73, 74).

## Conflict of interest

The authors declare that they have no conflicts of interest with the contents of this article.
