# Supplementary material for: The RsfSR two-component system regulates SigF function by monitoring the state of the respiratory electron transport chain in Mycobacterium smegmatis
Source: J Biol Chem. 2024 Feb 16;300(3):105764. doi: 10.1016/j.jbc.2024.105764 (PMC10950880; doi:10.1016/j.jbc.2024.105764)
Supplement: Supporting Information [file mmc1.docx]

**The RsfSR two-component system regulates SigF function by monitoring the state of the respiratory electron transport chain in *Mycobacterium smegmatis***

Yuna Oh^1^and Jeong-Il Oh^1,2*^

^1^Department of Integrated Biological Science, Pusan National University, 46241 Busan, Korea

^2^Microbiological Resource Research Institute, Pusan National University, 46241 Busan, Korea

***Correspondence:**

Jeong-Il Oh

joh@pusan.ac.kr

**Running title**: Regulation of the SigF partner switching system

**Keywords:** CHASE3, gene regulation, mycobacteria, *Mycobacterium smegmatis*, partner switching system, respiration, respiratory chain, SigF, sigma factor, two-component system

**Experimental procedures**

**Construction of mutant strains of *M. smegmatis*** Deletion mutants of *M. smegmatis* were constructed by allelic exchange mutagenesis using the suicide vector pKOTs containing a temperature-sensitive replication origin as described previously (70). In brief, the temperature-sensitive suicide plasmid was introduced into *M. smegmatis* by electroporation. Transformants were selected at 30℃ (replication-permissive temperature) on 7H9-glucose agar plates containing hygromycin, and the selected transformants were grown in 7H9-glucose liquid medium supplemented with hygromycin for 3-6 days at 30℃. Heterogenotes of *M. smegmatis*, which were generated by a single recombination event, were selected for their hygromycin resistance on 7H9-glucose agar plates at 42℃ (replication-nonpermissive temperature). The selected heterogenotes were grown on 7H9-glucose medium without antibiotics for 3-6 days at 37℃. Isogenic homogenotes were obtained from the heterogenotes after a second recombination by selecting them for sucrose resistance on 7H9-glucose agar plates containing 10% (w/v) sucrose at 37℃. The allelic exchange was verified by PCR with isolated genomic DNA (**Fig. S1**).

**(i) Δ*rsfR* and**Δ***aa*_3_**Δ***rsfR* mutants.** To construct the Δ*rsfR* andΔ*aa*_3_Δ*rsfR* mutants of *M. smegmatis*, the allelic exchange using pKOTsΔrsfR was performed in the WT and Δ*aa*_3_strains of *M. smegmatis*, respectively.

**(ii) Δ*rsfS* and**Δ***aa*_3_**Δ***rsfS* mutants.** To construct the Δ*rsfS* andΔ*aa*_3_Δ*rsfS* mutants of *M. smegmatis*, the allelic exchange using pKOTsΔrsfS was performed in the WT and Δ*aa*_3_strains of *M. smegmatis*, respectively.

**(iii) Δ*6128* and**Δ***aa*_3_**Δ***6128* mutants.** To construct the Δ*6128* andΔ*aa*_3_Δ*6128* mutants of *M. smegmatis*, the allelic exchange using pKOTsΔ6128 was performed in the WT and Δ*aa*_3_ strains of *M. smegmatis*, respectively.

**(iv) Δ*bc*_1_mutants.** To construct the Δ*bc*_1_mutant of *M. smegmatis*, the allelic exchange using pKOTsΔqcrCAB was performed in the WTstrain of *M. smegmatis*.

**Construction of plasmids**

**(i) The temperature-sensitive suicide plasmids for the construction of mutant strains of *M. smegmatis.***

To construct pKOTsΔrsfR, PCR was conducted with the F_rsfR_mut and R_rsfR_mut primers and the chromosomal DNA of *M. smegmatis* mc^2^155as a template. The amplified 1,434-bp DNA fragment was restricted with HindIII and NotI and cloned into pBSII KS+ digested with the same enzymes, yielding pBSIIrsfR. The 480-bp DNA fragment within *rsfR* was excised from pBSIIrsfR by restriction with BamHI, and the linear plasmid was self-ligated, resulting in pBSIIΔrsfR. A 943-bp HindIII-NotI DNA fragment from pBSIIΔrsfR was cloned into pKOTs, resulting in pKOTsΔrsfR.

To construct pKOTsΔrsfS, PCR was conducted with the F_rsfS_mut and R_rsfS_mut primers and the chromosomal DNA of *M. smegmatis* mc^2^155 as a template. The amplified 2,016-bp DNA fragment was restricted with BamHI and HindIII and cloned into pUC19 digested with the same enzymes, yielding pUCrsfS. The 840-bp DNA fragment within *rsfS* was excised from pUCrsfS by restriction with SacII, and the linear plasmid was self-ligated, resulting in pUCΔrsfS. An 1,156-bp DNA fragment was amplified by PCR reaction with the primers, F_rsfS_mut and R_rsfS_mut, and pUCΔrsfS as a template. The amplified PCR product was restricted with HindIII and cloned into pKOTs digested with EcoRV and HindIII, yielding pKOTsΔrsfS.

For the construction of pKOTsΔ6128, two rounds of recombination PCR were conducted. Using the chromosomal DNA of *M. smegmatis* mc^2^155 as a template, two primary PCR reactions were performed with the primers F_6128_mut and R_6128_rec, as well as with the primers F_6128_rec and R_6128_mut to generate two 39-bp overlapping DNA fragments (459 and 418 bp, respectively). Both PCR products contain the same 366-bp deletion within *MSMEG_6128* in the overlapping region. In the secondary PCR, an 838-bp DNA fragment with in-frame deletion of *MSMEG_6128* was obtained using both the primary PCR products as templates and the F_6128_mut and R_6128_mut primers. The secondary PCR product was restricted with NotI and HindIII and cloned into pKOTs digested with the same enzymes, yielding pKOTsΔ6128.

For the construction of pKOTsΔqcrCAB, two rounds of recombination PCR were conducted. Using the chromosomal DNA of *M. smegmatis* mc^2^155 as a template, two primary PCR reactions were performed with the primers F_bc1_mut and R_bc1_rec, as well as with the primers F_bc1_rec and R_bc1_mut to generate two 38-bp overlapping DNA fragments (408 and 356 bp, respectively). Both PCR products contain the same 3313-bp deletion within the *qcrCAB* operonin the overlapping region. In the secondary PCR, a 785-bp DNA fragment with deletion of *qcrCAB* was obtained using both the primary PCR products as templates and the F_bc1_mut and R_bc1_mut primers. The secondary PCR product was restricted with NotI and HindIII and cloned into pKOTs digested with the same enzymes, yielding pKOTsΔqcrCAB.

**(ii) pT7-7rsfR, pT7-7MSMEG6128, and pETrsfSTr.**

A 1,244-bp DNA fragment encompassing the *rsfR* gene and six His codons immediately before its stop codon was amplified by PCR with the primers F_rsfR_over and R_rsfR_over, using the chromosomal DNA of *M. smegmatis* mc^2^155 as a template and *Pfu* DNA polymerase. The PCR product was restricted with NdeI and pstI and cloned into pT7-7, yielding pT7-7rsfR.

A 482-bp DNA fragment encompassing the *MSMEG_6128* gene and six His codons immediately before its stop codon was amplified by PCR with the primers F_6128_over and R_6128_over, using the chromosomal DNA of *M. smegmatis* mc^2^155 as a template and *Pfu* DNA polymerase. The PCR product was restricted with NdeI and BamHI and cloned into pT7-7, yielding pT7-7rsfS.

A 1,046-bp DNA fragment encompassing the truncated *rsfS* gene and six His codons immediately before its stop codon was amplified by PCR with the primers F_rsfSTr_over and R_rsfS_over, using the chromosomal DNA of *M. smegmatis* mc^2^155 as a template and *Pfu* DNA polymerase. The PCR product was restricted with NdeI and BamHI and cloned into pET29b, yielding pETrsfSTr.

**(iii) pT7-7rsfRD74A and pT7-7rsfRD74E**

To introduce point mutations (D74A and D74E) into RsfR, PCR-based site-directed mutagenesis was performed using pT7-7rsfR as a template and the primers listed in Table S2.

**(iv) pMVRsfR**

For the construction of pMVRsfR, a 1,413-bp DNA fragment containing the *rsfR* gene was amplified by PCR with the F_rsfR_comp and R_rsfR_comp primers and the chromosomal DNA of *M. smegmatis* mc^2^155 as a template. The PCR product was restricted with XbaI and HindIII and cloned into pMV306, resulting in pMVRsfR.

**(v) pMHRsfS and pMHRsfSTr**.

For the construction of pMHRsfS, a 1,622-bp DNA fragment containing the *rsfS* gene was amplified by PCR with the F_rsfS_over and R_rsfS_2B8 primers and the chromosomal DNA of *M. smegmatis* mc^2^155 as a template. The PCR product was restricted with NdeI and ClaI and cloned into pMH201, resulting in pMHRsfS.

For the construction of pMHRsfSTr, a 1,055-bp DNA fragment containing the truncated *rsfS* gene was amplified by PCR with the F_rsfSTr_over and R_rsfS_2B8 primers and the chromosomal DNA of *M. smegmatis* mc^2^155 as a template. The PCR product was restricted with NdeI and ClaI and cloned into pMH201, resulting in pMHRsfSTr.

**(vi) pMVRsfSAla and pMHRsfSAla.**

To introduce five alanine codons into the region of *rsfS* encoding the CHASE3 domain, two rounds of recombination PCR were conducted. Using the chromosomal DNA of *M. smegmatis* mc^2^155 as a template, two primary PCR reactions were performed with the F_rsfS_comp and R_rsfS_ala primers, as well as with the F_rsfS_ala and R_rsfS_2B8 primers to generate two 39-bp overlapping DNA fragments (397 and 1,290 bp, respectively). The two primary PCR products were used as templates for the secondary PCR, which was performed using primers F_rsfS_comp and R_rsfS_2B8. In the secondary PCR, a 1,661-bp DNA fragment with the insertion mutation of *rsfS* was obtained using both the primary PCR products as templates and the F_rsfS_comp and R_rsfS_2B8 primers. The secondary PCR product was restricted with NcoI and HindIII and cloned into pMV306 digested with the same enzymes, yielding pMVRsfSAla.

For the construction of pMHRsfSAla, a 1,636-bp DNA fragment containing the mutant form of *rsfS* gene was amplified by PCR with the F_rsfS_over and R_rsfS_2B8 primers and pMVRsfSAla as a template. The PCR product was restricted with NdeI and ClaI and cloned into pMH201, resulting in pMHRsfSAla.


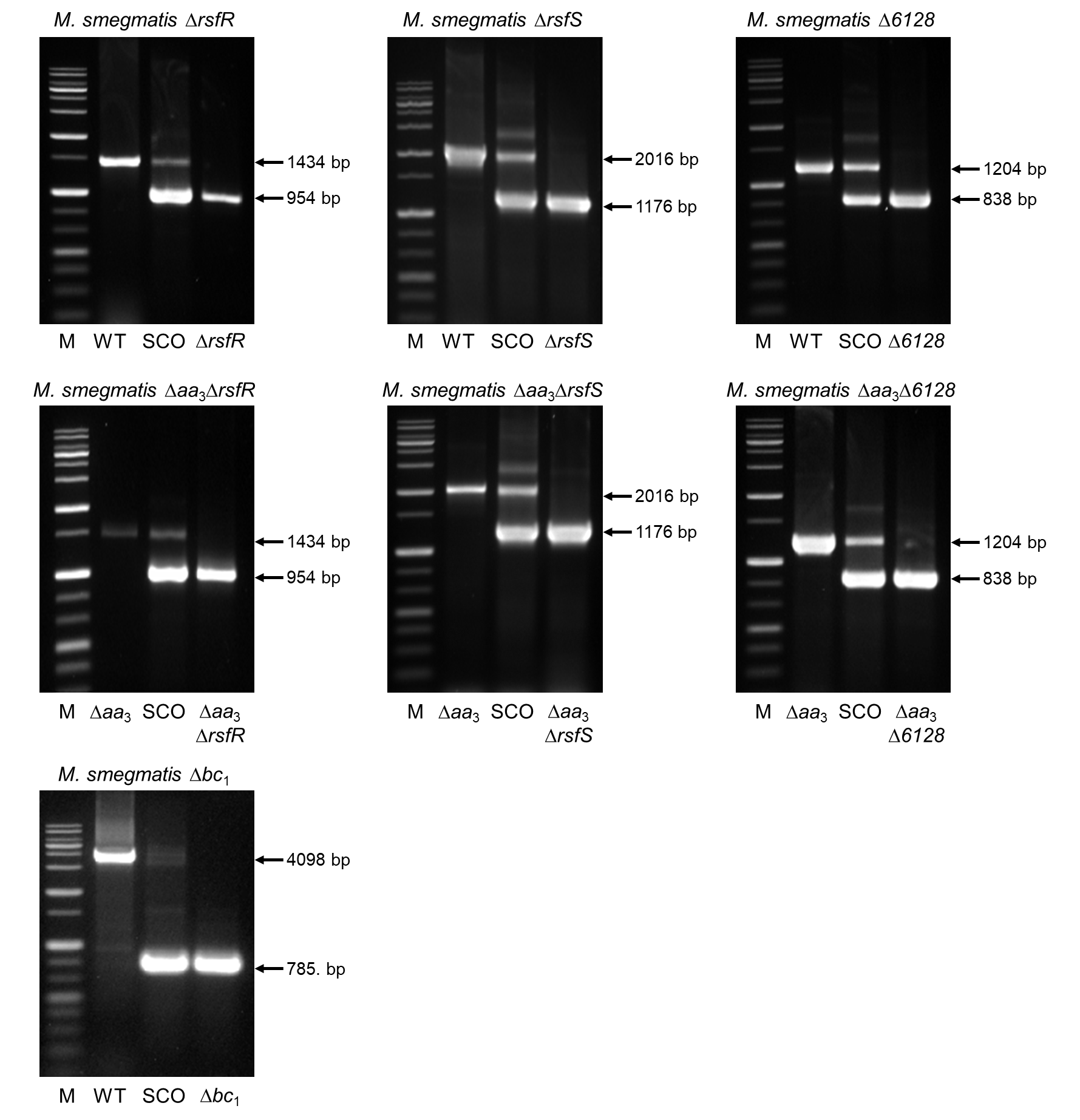


**Figure S1.** **Validation of the Δ*rsfS,* Δ*rsfR,* Δ*6128,* Δ*aa*_3_Δ*rsfS*, Δ*aa*_3_Δ*rsfR*, Δ*aa*_3_Δ*6128,* andΔ*bc*_1_ deletion mutants of *M. smegmatis* by PCR.** The PCR reactions were performed with the gene-specific primers using the chromosomal DNA from the WT,** **Δ*aa*_3_, single crossover (SCO), and deletion mutant strains. The PCR products are indicated by the arrows with their expected size. The primer sets employed for confirmation of deletions are listed in Table S2. M, DNA size marker lanes**.**

**
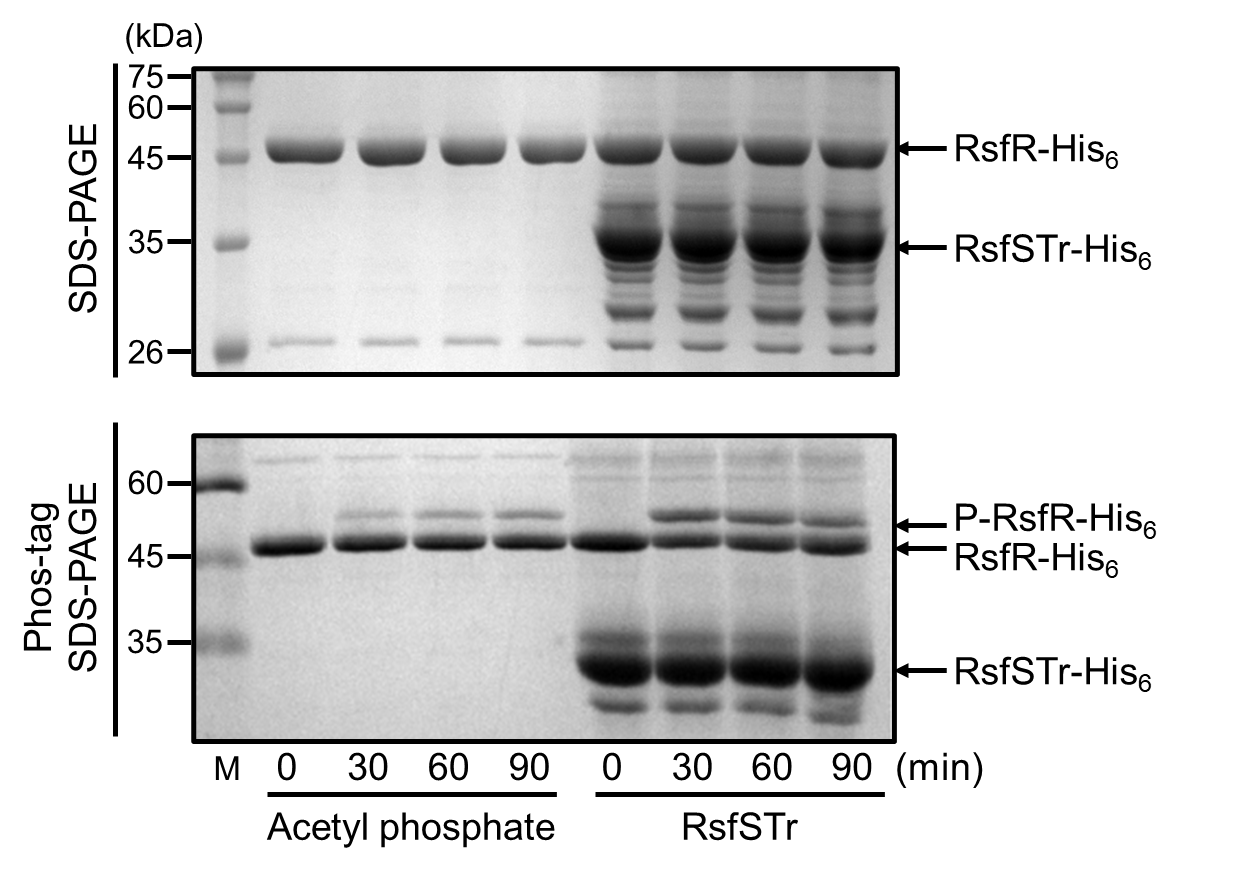
**

**Figure S2.** **Phosphorylation of RsfR by acetyl phosphate and RsfSTr.**

For *in vitro* phosphorylation assay using purified RsfR and acetyl phosphate (Acetyl phosphate), 154 pmol of purified RsfR was incubated in the reaction mixture [300 mM Tris-Cl (pH 8.0), 50 mM KCl, and 10 mM MgCl_2_] containing 40 mM acetyl phosphate (the total reaction volume is 22 µl) at 30℃. For *in vitro* phosphorylation assay using purified RsfR and RsfSTr (RsfSTr), 550 pmol of partially purified RsfSTr was autophosphorylated in the reaction mixture with 1 mM ATP for 30 min at 30℃. Following the addition of 154 pmol of purified RsfR to the autophosphorylation reaction mixture (the total reaction volume is 22 µl), the phosphotransfer reactions were performed at 30℃. The reactions were stopped at the indicated time points by the addition of 11 µl of 3 x gel-loading buffer. 15 µl each of the stopped reactions was subjected to SDS-PAGE (upper gel) and 75 µM Mn^2+^-Phos-tag SDS-PAGE (lower gel).The gels were stained with CBB. The bands of His_6_-tagged RsfSTr (RsfSTr-His_6_), unphosphorylated RsfR (RsfR-His_6_), and phosphorylated RsfR (P-RsfR-His_6_) are indicated by the arrows. M, molecular weight marker lanes.

**Table S1.** Thestrains and plasmids used in this study

| **Strain/plasmid** | **Relevant phenotype/genotype^a^** | **Reference** |
| --- | --- | --- |
| **Strains** |  |  |
| *E. coli* DH5α | φ80dl*acZ*ΔM15 Δ*lacU169* *recA1* *endA1* *hsdR17* *supE44 thi1 gyrA96 relA1* | (71) |
| *E. coli* BL21 (DE3) | F^-^ *ompT hsd*S*_B_* (r_B_^-^ m_B_^-^) *dcm gal* λ (DE3) | Promega |
| *M. smegmatis* mc^2^155 | High-transformation-efficiency mutant of *M. smegmatis* ATCC 607 | (63) |
| *M. smegmatis* Δ*rsfR* | *MSMEG_6131* (*rsfR*)deletion mutant derived from *M*. *smegmatis* mc^2^155 | This study |
| *M. smegmatis* Δ*rsfS* | *MSMEG_6130* (*rsfS)* deletion mutant derived from *M. smegmatis* mc^2^155 | This study |
| *M. smegmatis* Δ*6128* | *MSMEG_6128* deletion mutant derived from *M*. *smegmatis* mc^2^155 | This study |
| *M. smegmatis* Δ*aa*_3_ | *MSMEG_4268* (*ctaC*)deletion mutant derived from *M*. *smegmatis* mc^2^155 | (31) |
| *M. smegmatis* Δ*aa*_3_Δ*rsfS* | *MSMEG_4268* (*ctaC*) and *MSMEG_6130* (*rsfS*)double-deletion mutant derived from *M*. *smegmatis* mc^2^155 | This study |
| *M. smegmatis* Δ*aa*_3_Δ*rsfR* | *MSMEG_4268* (*ctaC*) and *MSMEG_6131* (*rsfR*)double-deletion mutant derived from *M*. *smegmatis* mc^2^155 | This study |
| *M. smegmatis* Δ*aa*_3_Δ*6128* | *MSMEG_4268* (*ctaC*) and *MSMEG_6128* double-deletion mutant derived from *M*. *smegmatis* mc^2^155 | This study |
| *M. smegmatis* Δ*bd* | *MSMEG_3233(cydA)* deletion mutant derived from *M*. *smegmatis* mc^2^155 | (31) |
| *M. smegmatis* Δ*bc*_1_ | *MSMEG_4261* (*qcrC*), *MSMEG_4262* (*qcrA*) and *MSMEG_4263* (*qcrB*) deletion mutant derived from *M*. *smegmatis* mc^2^155 | This study |
|  |  |  |
| **Plasmids** |  |  |
| pT7-7 | Amp^r^; T7 promoter, ribosome binding site, and translation start codon overlapping with NdeI site | (72) |
| pET29b | Km^r^, T7 promoter, ribosome binding site, and translation start codon overlapping with NdeI site | Novagen |
| pBSII KS+ | Amp^r^; *lacPOZ’* | Stratagene |
| pUC19 | Amp^r^; *lacPOZ’* | (73) |
| pKOTs | Hyg^r^; pKO-based vector constructed by inserting HindIII-KpnI fragment containing pAL500Ts and the pUC ori derived from pDE | (70) |
| pMV306 | Km^r^; integration vector containing *int* and *attP* site of mycobacteriophage L5 for integration into the mycobacterial genome | (74) |
| pMH201 | Km^r^; acetamide-inducible promoter, derivative of pMV306 | (75) |
| pNCII1777 | pNCII::0.456-kb XbaI-BamHI fragment containing the *MSMEG_1777* promoter region | (22) |
| pT7-7rsfB | pT7-7::0.384-kb NdeI-HindIII fragment containing *rsfB* (*MSMEG_6127*) with six His codons before its stop codons | (22) |
| pT7-7rsfR | pT7-7::1.233-kb NdeI-PstI fragment containing *rsfR* (*MSMEG_6131*) with six His codons before its stop codons | This study |
| pT7-7rsfRD74A | pT7-7rsfR in which the codon for Asp-74 (GAC) is replaced with GCG | This study |
| pT7-7rsfRD74E | pT7-7rsfB in which the codon for Asp-74 (GAC) is replaced with GAG | This study |
| pT7-7MSMEG6128 | pT7-7::0.471-kb NdeI-BamHI fragment containing *MSMEG_6128* with six His codons before its stop codons | This study |
| pETrsfSTr | pET29b::1.035-kb NdeI-BamHI fragment containing the truncated *rsfS* (*MSMEG_6130*) gene with six His codons before its stop codons | This study |
| pUCrsfS | pUC19::2.006-kb BamHI-HindIII fragment containing *rsfS* | This study |
| pUCΔrsfS | pUC19::1.166-kb BamHI-HindIII fragment containing Δ*rsfS* (*MSMEG_6130*) | This study |
| pKOTsΔrsfS | pKOTs::1.171-kb EcoRV-HindIII fragment containing Δ*rsfS* (*MSMEG_6130*) | This study |
| pBSIIrsfR | pKOTs::1.423-kb HindIII-NotI fragment containing Δ*rsfR* (*MSMEG_6131*) | This study |
| pBSIIΔrsfR | pKOTs::0.943-kb HindIII-NotI fragment containing Δ*rsfR* (*MSMEG_6131*) | This study |
| pKOTsΔrsfR | pKOTs::0.943-kb HindIII-NotI fragment containing Δ*rsfR* (*MSMEG_6131*) | This study |
| pKOTsΔ6128 | pKOTs::0.827-kb NotI-HindIII fragment containing Δ*6128* (*MSMEG_6128*) | This study |
| pKOTsΔqcrCAB | pKOTs::0.774-kb NotI-HindIII fragment containing Δ*bc*_1_ | This study |
| pMVRsfR | pMV306::1.403-kb XbaI-HindIII fragment containing *rsfR* (*MSMEG_6131*) | This study |
| pMVRsfRD74A | pMVRsfR in which the codon for Asp-74 (GAC) is replaced with GCG | This study |
| pMVRsfSAla | pMV306::1.651-kb NcoI-HindIII fragment containing *rsfS* (*MSMEG_6130*) with five Ala codons insertion | This study |
| pMHRsfB | pMH201::0.387-kb NdeI-ClaI fragment containing C-terminally His_6_-tagged *rsfB* (*MSMEG_6127*) | (22) |
| pMHRsfS | pMH201::1.610-kb NdeI-ClaI fragment containing *rsfS* (*MSMEG_6130*) | This study |
| pMHRsfSTr | pMH201::1.043-kb NdeI-ClaI fragment containing the truncated *rsfS* gene | This study |
| pMHRsfSAla | pMH201::1.625-kb NdeI-ClaI fragment from containing *rsfS* (*MSMEG_6130*) with finsertion of five Ala codons | This study |

*Abbreviations: Amp^r^, ampicillin resistance; Cam^r^, chloramphenicol resistance; Hyg^r^, hygromycin resistance; Km^r^, kanamycin resistance. Str^r^, streptomycin resistance Tet^r^, tetracycline resistance

**Table S2.** The oligonucleotides used in this study

| **Oligonucleotide** | **Nucleotide sequences (5'→3')** | **Purpose** |
| --- | --- | --- |
| F_rsfR_mut | ATTAAAGCTTGTTTGCAGTCCACACGTCG | Δ*rsfR* construction and confirmation |
| R_rsfR_mut | ATTA AAGCTTGACCGACAGAACCAGGTTC | Δ*rsfR* construction and confirmation |
| F_rsfS_mut | ATTAGGATCCGTTGCTGCTGTTGACCGAC | Δ*rsfS* construction and confirmation |
| R_rsfS_mut | ATTAAAGCTTTCGAGCAGAATCAGGTCC | Δ*rsfS* construction and confirmation |
| F_6128_mut | ATTAGCGGCCGCGATCATGGTGTGGCAGAACC | Δ*6128* construction and confirmation |
| R_6128_rec | CACCACCTGGACGAAGAACTCCAGGAGGATGTCGATCGC | Δ*6128* construction |
| F_6128_rec | GCGATCGACATCCTCCTGGAGTTCTTCGTCCAGGTGGTG | Δ*6128* construction |
| R_6128_mut | ATTAAAGCTTGAATTCTCCGAATGGCTC | Δ*6128* construction and confirmation |
| F_bc1_mut | ATTAGCGGCCGCGTCATCACCTTCCTCATGG | Δ*bc*_1_ construction and confirmation |
| R_bc1_rec | CTGGTGCAGCTCGACGTAACCGATCAGCAGCAGAAG | Δ*bc*_1_ construction |
| F_bc1_rec | CTTCTGCTGCTGATCGGTTACGTCGAGCTGCACCAG | Δ*bc*_1_ construction |
| R_bc1_mut | ATTAAAGCTTGGGAGTGGCGCATATAGAAAG | Δ*bc*_1_ construction and confirmation |
| F_rsfR_comp | ATTAAAGCTTGTTTGCAGTCCACACGTCG | Δ*aa*_3_Δ*rsfR* complementation |
| R_rsfR_comp | ATTAAAGCTTTCAGTGATGGTGATGGTGATGTCTGCGCGAGCGCTC | Δ*aa*_3_Δ*rsfR* complementation |
| F_rsfS_over | ATTACATATGAGATTGACGGTACAA | RsfS overexpression |
| F_rsfSTr_over | ATTACATATGATCGTGGCGTTCTTCGCA | RsfSTr overexpression |
| R_rsfS_over | ATTAGGATCCTCAGTGATGGTGATGGTGATGTGTGTTGTTTCCTTC | RsfSTr overexpression |
| F_rsfS_comp | ATTACCATGGATGACATCGCCGTGATAC | RsfSAla overexpression |
| R_rsfS_ala | ATCACGGCGAGGGCCGCGGCCGCCGCATCGGCGAGGAGTTCGTCGCG | RsfSAla overexpression |
| F_rsfS_ala | TCCTCGCCGATGCGGCGGCCGCGGCCCTCGCCGTGATCGAAAAGGCT | RsfSAla overexpression |
| R_rsfS_2B8 | TAATATCGATTCAGGGGGGGAAGGCGGGCAGCGGGTCGCGTGTGTTGTTTCCTTCC | RsfS, RsfSTr, and RsfSAla overexpression |
| F_rsfR_over | ATTACATATGCGCGCGCCATTTGAC | RsfRoverexpression |
| R_rsfR_over | ATTACTGCAGTCAGTGATGGTGATGGTGATGTCTGCGCGAGCGCTC | RsfRoverexpression |
| F_6128_over | ATTACATATGACGTCAGCCGACGAA | MSMEG_6128overexpression |
| R_6128_over | ATTAGGATCCTCAGTGATGGTGATGGTGATGGAACTGCGGTAACCG | MSMEG_6128overexpression |
| F_RsfR_D74A | GACTGTGTCCTGCTCGCGCTCAATCTTCCCGAT | *rsfB* point mutation |
| R_RsfR_D74A | ATCGGGAAGATTGAGCGCGAGCAGGACACAGTC | *rsfB* point mutation |
| F_RsfR_D74E | GACTGTGTCCTGCTCGAGCTCAATCTTCCCGAT | *rsfB* point mutation |
| R_RsfR_D74E | ATCGGGAAGATTGAGCTCGAGCAGGACACAGTC | *rsfB* point mutation |

‘
